# Foxn1 Regulates Lineage Progression in Cortical and Medullary Thymic Epithelial Cells But Is Dispensable for Medullary Sublineage Divergence

**DOI:** 10.1371/journal.pgen.1002348

**Published:** 2011-11-03

**Authors:** Craig S. Nowell, Nicholas Bredenkamp, Stéphanie Tetélin, Xin Jin, Christin Tischner, Harsh Vaidya, Julie M. Sheridan, Frances Hogg Stenhouse, Raphaela Heussen, Andrew J. H. Smith, C. Clare Blackburn

**Affiliations:** MRC Centre for Regenerative Medicine, Institute for Stem Cell Research, School of Biological Sciences, University of Edinburgh, Edinburgh, United Kingdom; The Jackson Laboratory, United States of America

## Abstract

The forkhead transcription factor *Foxn1* is indispensable for thymus development, but the mechanisms by which it mediates thymic epithelial cell (TEC) development are poorly understood. To examine the cellular and molecular basis of Foxn1 function, we generated a novel and revertible hypomorphic allele of *Foxn1*. By varying levels of its expression, we identified a number of features of the Foxn1 system. Here we show that Foxn1 is a powerful regulator of TEC differentiation that is required at multiple intermediate stages of TE lineage development in the fetal and adult thymus. We find no evidence for a role for Foxn1 in TEC fate-choice. Rather, we show it is required for stable entry into both the cortical and medullary TEC differentiation programmes and subsequently is needed at increasing dosage for progression through successive differentiation states in both cortical and medullary TEC. We further demonstrate regulation by Foxn1 of a suite of genes with diverse roles in thymus development and/or function, suggesting it acts as a master regulator of the core thymic epithelial programme rather than regulating a particular aspect of TEC biology. Overall, our data establish a genetics-based model of cellular hierarchies in the TE lineage and provide mechanistic insight relating titration of a single transcription factor to control of lineage progression. Our novel revertible hypomorph system may be similarly applied to analyzing other regulators of development.

## Author Summary

The thymus is the specialized organ responsible for generating T cells, which are required to regulate and effect immune responses. The unique functions of the thymus are mediated by a diverse array of specialized epithelial cells found only within this organ. These specialized, functionally mature thymic epithelial cells are generated from immature epithelial progenitor cells present in the fetal and adult thymus through a highly regulated process, termed differentiation, that is tightly controlled by specific genes. Foxn1, a protein that is expressed in thymic epithelial cells, is a transcription factor—a protein that regulates how other genes are expressed. Here, we have investigated the role of Foxn1 in generating mature thymic epithelial cells from immature progenitors. We find that Foxn1 is required throughout this process, from the onset of differentiation in progenitor thymic epithelial cells in the developing fetus to the final differentiation steps through which thymic epithelial cells mature to acquire their full functionality. We further find that Foxn1 controls the expression of a variety of genes with different functions in thymic epithelial cells. Overall, our study defines the role of Foxn1 in thymus development at the cellular level and provides insight into how it mediates these functions.

## Introduction

T cell development occurs in the thymus and depends on progressive interactions with the thymic stroma. Critical to this function is the thymic epithelium (TE), which comprises a diverse array of phenotypically and functionally distinct cell types broadly organized into cortical and medullary regions [1].

During development the thymus arises from the endoderm of the third pharyngeal pouches [2] which, from day 9 of embryonic development in the mouse (E9.0), contain cells specified to the TE lineage [2]. Until E11.5, this region uniformly expresses the cell surface determinant Plet-1 [3]-[5] and the earliest currently identified founder cells for the lineage are thus Plet-1^+^ third pharyngeal pouch cells. By E12.5 the thymic primordia, now colonized by haematopoietic progenitor cells and surrounded by a mesenchymal capsule, have separated from the pharyngeal endoderm and contain a predominant EpCam^+^Plet1^+^ epithelial population [3], [6], [7]. Recent evidence supports the existence within this population of a common thymic epithelial progenitor cell (TEPC) capable of generating both cortical and medullary TEC (cTEC and mTEC respectively) [7], [8]. Further studies indicate that a medullary sub-lineage specific progenitor cell is present at least as early as E13.5 [9], [10], and suggest the existence of a cortical sub-lineage specific progenitor [11]. However, the extent to which these progenitor activities persist in late organogenesis and the postnatal organ has not been determined and furthermore, the timing of emergence of the sub-lineage progenitors during organogenesis remains unclear.

The forkhead transcription factor Foxn1 [12], the gene mutated in the classical mouse mutant *nude* (*nu)* [13], is a pivotal regulator of TE lineage development. Nude mice are hairless and athymic [14], [15]. In *Foxn1* null animals, the earliest stages of thymus development appear to occur normally, but development is arrested after initial formation of the organ primordium (around E12.0 in the mouse) and the primordium is not colonized by hematopoietic precursors [16]. Nonetheless, the *Foxn1* null thymic primordium separates from the parathyroid component of the common primordium and migrates to the mid-line, where a small, alymphoid cystic thymic remnant persists postnatally [14]. Null alleles of *Foxn1* in mouse, rat and human all exhibit essentially the same pleiotropic phenotype [17].

Forkhead family members play important roles in specification and progression of a number of cell lineages, and recent evidence reveals roles for some Forkhead proteins in chromatin modification/epigenetic regulation of cell fate [18]. Analysis of postnatal *nude*:wild-type chimeric mice demonstrated the cell autonomous requirement for Foxn1 in development of all major TEC sub-lineages and suggested that TE lineage cells unable to express functional Foxn1 might undergo maturational arrest at a common TEPC stage [19]. A revertible *Foxn1* null model, *Foxn1^SA2/SA2^* provided strong support for this hypothesis, as neonatal clonal reversion of this allele resulted in the generation of small units of functional thymus tissue containing both cortical and medullary compartments [8]. Further support for a role in differentiation comes from studies on keratinocytes, which implicate *Foxn1* in regulating initiation of terminal differentiation [20], [21], and from analysis of a hypomorphic *Foxn1* allele which generates a transcript lacking exon 3 and thus the N-terminal domain of Foxn1 [22]. In mice homozygous for this allele (*Foxn1^Δ^*), the postnatal *Foxn1^Δ/Δ^* thymus was highly cystic, contained no discernable cortical or medullary regions and could sustain only highly impaired thymocyte differentiation, suggesting that Foxn1 is actively required for TEC differentiation at stages beyond initiation of the TEC programme [22]. Evidence also supports roles for Foxn1 in TEC proliferation [23] and in regulating the balance between proliferation and differentiation in skin [24]. In addition, a requirement for Foxn1 for maintenance of the postnatal thymic microenvironment has recently been demonstrated [25]–[27], with evidence pointing to differential sensitivity of different TEC subsets to changes in Foxn1 dosage [26]. Collectively, these studies suggest that Foxn1 plays a complex role in regulating TEC lineage development. However, precisely how Foxn1 regulates the transit from the earliest fetal thymic epithelial progenitor cell to the fully functional postnatal thymic epithelium, and at which stages in this process it is required, remains undetermined. In addition, the molecular mechanisms regulated by this transcription factor in the thymus have not yet been addressed.

In this study, we have addressed the functions of Foxn1 throughout thymus ontogeny, via generation and analysis of a novel revertible hypomorphic allele of *Foxn1, Foxn1^R^,* which expresses only low levels of Foxn1 mRNA and protein. A particular advantage of our system is the revertible nature of the allele, which affords the capacity to test the relationship of cell states identified via analysis of mutant mice to states occurring in normal ontogeny. Our studies establish Foxn1 as a powerful regulator of differentiation in both the cTEC and mTEC sub-lineages. We find no evidence for a role for Foxn1 in regulating cell fate choice in the cortical or medullary TEC sub-lineages. Rather, we find that Foxn1 is required for progression of differentiation at multiple stages in cTEC and mTEC sub-lineage development in both the fetal and postnatal thymus, and show that different Foxn1 dosage is required to execute its function(s) at different differentiation stages. We further establish that Foxn1 regulates, either directly or indirectly, a suite of genes known to effect TEC function - including *Dll4*, *CCL25*, *Cathepsin L, CD40, Pax1*, and *MHC Class II*, and that these exhibit different response patterns to changes in Foxn1 dosage. Collectively, these findings significantly advance understanding of the role of Foxn1 in TEC, demonstrating for the first time its direct involvement in generating multiple TEC sub-types in both the cortical and medullary lineages in the fetal and postnatal thymus, and suggesting it acts effectively as a master regulator of the entire TEC programme.

## Results

### Generation of a revertible hypomorphic allele of Foxn1

**Figure 1:**
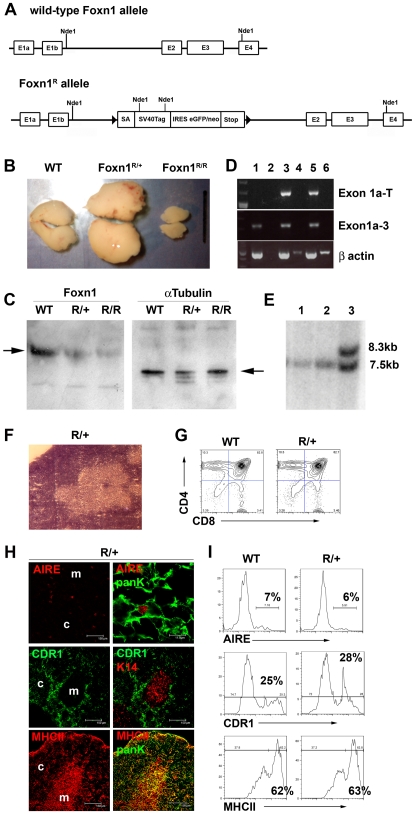
*Foxn1^R^* is a hypomorphic allele. (A) A LoxP flanked cassette containing the 5′ engrailed 2 splice acceptor site (SA), the SV40TAg coding region, an internal ribosome entry site coupled to an enhanced green fluorescent protein/neomycin resistance fusion protein (IRES-EGFPneo), and the CMAZ transcriptional pause [65] was inserted into intron 1b of the *Foxn1* locus of mouse ES cells (Figure S1). E, exon. (B) Images show representative pairs of thymic lobes from six week old WT, *Foxn1^R/+^* and *Foxn1^R/R^* as indicated. Scale bars represent 1cm. Thymocyte numbers - *Foxn1^R/R^*, 5.0×10^7^±2.3×10^6^; *Foxn1^R/+^*, 2.6x10^9^±9.5×10^7^; WT littermates 1.9×10^8^ ±8.1×10^6^. WT versus *Foxn1^R/+^* p = 3.7×10^−6^; WT versus *Foxn1^R/R^* p = 2.0×10^−5^, n = 3 for each genotype. TEC numbers: WT, 1.3×10^6^±8.4×10^5^; *Foxn1^R/+^*, 1.3×10^7^±1.8×10^6^; *Foxn1^R/R^*, 3.6×10^5^±2.1×10^5^. WT versus *Foxn1^R/+^* p = 0.00054; WT versus *Foxn1^R/R^* p = 0.09374. (C) Immunoblots performed on nuclear extracts isolated from whole E12.5 thymus tissue were probed sequentially with anti-Foxn1 (left panel) and anti-alpha-tubulin (loading control; right panel). Arrows indicate bands corresponding to Foxn1 and alpha-tubulin. Genotypes are indicated above each image. WT, wild-type; R/+, *Foxn1^R/+^*; R/R, *Foxn1^R/R^*. (D) RT-PCR analysis of *Foxn1^R/R^* TEC reveals splicing around the inserted cassette. cDNA from whole adult thymus from wild type, *Foxn1^R/+^* or *Foxn1^R/R^* thymi was subjected to RT-PCR using primers specific for *Foxn1* exon1a (forward) and exon 3 (reverse); *Foxn1* exon1a (forward) and *SV40Tag* (reverse), or *ß actin* as shown. Lanes: m, markers; 1, wild type cDNA; 2, wild type no RT control; 3, *Foxn1^R/+^* cDNA; 4, *Foxn1^R/+^* no RT control; 5, *Foxn1^R/R^* cDNA; 6, *Foxn1^R/R^* no RT control. Bands in lanes 4 and 6 of the ß actin panel are non-specific. Band specificity was confirmed by sequencing. (E) Reversion of the *Foxn1^R^* allele in *Foxn1^R/+^ZP3^Cre/+^* mice. *Foxn1^R/+^* males were mated with *ZP3^Cre/Cre^* females to generate *Foxn1* ^R/+:^ *ZP3* ^Cre/+^ mice. *Foxn1^R/+:^ZP3^Cre/+^* females were then crossed with C57BL/6 males and the resulting offspring analyzed. Two mice (lanes 1 and 2) were identified by PCR as carrying both the *Foxn1^R^* allele (using primers specific for the targeted allele) and the *ZP3^Cre^* transgene and were subsequently analyzed by Southern blotting of Nde1 digested genomic DNA using a probe flanking the 3′ end of the homology arms. In both cases only a 7.5kb band was present indicating complete excision of the targeted insertion. Lane 3 shows the 8.5kb and 7.5kb bands corresponding to the targeted and WT alleles respectively in a Cre negative *Foxn1^R/+^* littermate. (F-I) Morphological and phenotypic analysis of 6 week-old *Foxn1^R/+^* thymi. (F) Representative H&E staining showing overtly normal architecture in *Foxn1^R/+^* thymi. Magnification, x10. (G, I) Plots show flow cytometric analysis of 6 week old *Foxn1^R/+^* or WT thymi with markers shown. Plots in I) show data after gating against CD45^+^ and on EpCam^+^ cells. (H) Immunohistochemical analysis showing normal staining patterns with markers specific for medullary (K14, Aire) and cortical (CDR1) TEC and for the TEC maturation marker MHC Class II (MHCII). Epithelial cells are identified by co-staining with anti-pancytokeratin (PanK) in lower and upper panels. Right panels show co-stains of left panels, right AIRE panel shows detail from left panel. Scale bars 150 µm except right Aire panel which is 11.9 µm. Data shown in F-I are representative of at least three independent experiments. Thymocyte (G) and TEC (H,I) subset distribution is indistinguishable between *Foxn1^R/+^* and WT thymi.

We set out to generate a conditionally revertible null allele of *Foxn1* as a tool for generating TEPC lines. Our rationale was that lack of Foxn1 expression would impose an early block on TEC lineage differentiation, effectively trapping TEC in an undifferentiated progenitor cell state, while reversion of the allele would remove this block and allow progression to terminal differentiation. Since the extent to which Foxn1 is required for TEC proliferation is unknown, SV40 T antigen was used to uncouple potential roles of Foxn1 in proliferation and differentiation. We thus generated the revertible *Foxn1* allele, *Foxn1^R^*, by inserting a LoxP flanked cassette into intron 1b of the *Foxn1* locus by homologous recombination in ES cells (Figure 1A, Figure S1) and used this ES line to generate the *Foxn1^R^* mouse strain. Initial characterization of postnatal *Foxn1^R/+^* mice revealed thymus hyperplasia as expected, due to expression of SV40 Tag under the *Foxn1* promoter. However, *Foxn1^R/R^* mice developed severely hypoplastic thymi rather than exhibiting the expected phenotype of complete thymic aplasia (Figure 1B). In keeping with this, immunoblotting for Foxn1 revealed low-level Foxn1 protein in *Foxn1^R/R^* thymi compared to wild-type (WT; Figure 1C) and RT-PCR analyses demonstrated that the transcripts produced from the *Foxn1^R^* allele contained either Exon1a-SV40Tag-IRES-eGFPneo elements or the full-length *Foxn1* mRNA (Figure 1D). Thus some residual *Foxn1* expression from *Foxn1^R^* occurred, and resulted from splicing around the targeted insertion subsequent to transcription proceeding beyond the transcriptional pause. No overt skin phenotypes were apparent in either *Foxn1^R/+^* or *Foxn1^R/R^* mice. Collectively, these data establish that *Foxn1^R^* is a hypomorphic allele that expresses a low level of Foxn1 compared to WT and that expression of SV40Tag does not compensate for decreased levels of Foxn1.

The revertibility of the allele was demonstrated by crossing *Foxn1^R^* ^/+^ mice with the *ZP3^Cre^* deletor strain, in which Cre recombinase is expressed in oocytes [28]. *Foxn1^R/+^ZP3^Cre/+^* mice excised the LoxP flanked cassette in most if not all cells (Figure 1E), and showed normal thymus development and function.

### SV40 T antigen does not affect TEC differentiation or function in *Foxn1^R/+^* thymi

The Foxn1 null phenotype of severe athymia results from a complete early block in thymus development and has limited understanding of the role of Foxn1 later in ontogeny. We reasoned that the *Foxn1^R^* allele might be informative in this light. However, it was first necessary to test whether expression of SV40Tag under the *Foxn1* promoter affected TEC differentiation or function. Therefore, we characterized the *Foxn1^R/+^* thymus phenotype, in particular seeking evidence for impaired or perturbed TEC development and/or function. The two thymic lobes in *Foxn1^R/+^* mice exhibited a greater than ten-fold increase in both TEC and thymocyte numbers compared to WT littermates (Figure 1), but were otherwise histologically normal (Figure 1B, 1F). In both fetal and postnatal *Foxn1^R/+^* thymi, TEC and thymocyte subset distributions were indistinguishable from *Foxn1^nu/+^* and WT littermates and no evidence of malignant transformation could be found (Figure 1G-1I). We therefore concluded that the thymic hyperplasia observed resulted from a uniform expansion of the TEC compartment due to expression of SV40Tag under control of the *Foxn1* promoter, but that this did not affect TEC differentiation or function in either the fetal or postnatal thymus. This is consistent with the action of SV40TAg in a variety of other tissues [29]–[31] and its known function in regulating cell proliferation. Of note is that the *Foxn1* promoter drives relatively low levels of gene expression in TEC, based on QRT-PCR analyses relative to a range of housekeeping genes. This demonstrated that *Foxn1* is expressed in the same range as *alpha-tubulin*, which is recognized to be expressed at low levels [32] (Figure S2). Furthermore, SV40Tag was not detectable by immunohistochemical analysis of thymus sections from *Foxn1^R^* mutant mice (Figure S2).

### Terminal differentiation of postnatal cortical and medullary TEC is blocked in *Foxn1^R/R^* mice

**Figure 2:**
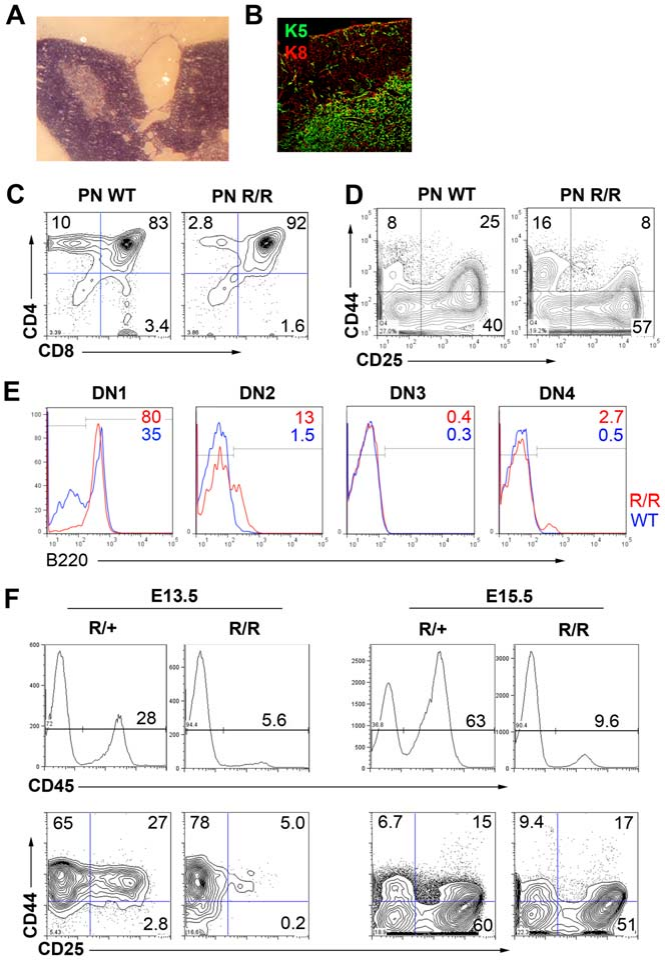
Functional deficit in *Foxn1^R/R^* TEC. Morphological and phenotypic analysis of 6 week old *Foxn1^R/R^* thymi. (A) H&E staining showing frequent large medullary cysts in *Foxn1^R/R^* thymi. (B) Immunostaining with anti-cytokeratin 5 (K5) and anti-cytokeratin 8 (K8) showing overtly normal cortical and medullary compartments in *Foxn1^R/R^* thymi; note the normal morphology of cortical and medullary TEC. (C-E) Flow cytometric analysis of 6 week old wild-type or *Foxn1^R/R^* thymocytes with (C) anti-CD4-PE, anti-CD8-FITC, (D) anti-CD44-APC and anti-CD25-PE after gating on Lin^-^ cells (Lin  =  anti-CD3, anti-CD4, anti-CD8, anti-CD11b, anti-CD11c, Ter119, NK1.1, Gr-1), or (E) B220 after gating on DN1 cells in (D). (E) red lines show *Foxn1^R/R^*; blue lines, WT. (F) Flow cytometric analysis of E13.5 or E15.5 fetal thymic primordia with anti-CD45-APC Cy7; anti-CD25-PE, and anti-CD44-APC. PN, postnatal; WT, wild type. (E) shows pooled data from three 6 week old mice, n = 1. All other data are representative of at least three separate analyses.

Next, we tested the effect of reduced Foxn1 dosage on postnatal TEC by analyzing the *Foxn1^R/R^* phenotype. Although small, *Foxn1^R/R^* thymi were grossly histologically normal, containing clear cortical and medullary regions (Figure 2A, 2B). Postnatal *Foxn1^R/R^* thymi contained all normal thymocyte subsets (Figure 2C, 2D). However, the CD4^+^8^+^ double positive (DP) subset was proportionally larger than in WT littermates while the CD4^+^ and CD8^+^ single positive (SP) subsets were proportionally reduced (DP proportions: *Foxn1^R/R^*, 91.2±0.85%; WT 82.9±0.21%, p = 0.0004) suggesting that the DP to SP transition and/or subsequent thymocyte maturation was perturbed in *Foxn1^R/R^* mice. The CD4^-^8^-^ (Double negative, DN) cell subset distribution was also altered in postnatal *Foxn1^R/R^* thymi, with increased proportions of CD44^+^CD25^-^ (DN1) and CD44^-^CD25^+^ (DN3) cells (Figure 2D). Most DN1 cells in postnatal *Foxn1^R/R^* thymi were B cells, as shown by B220 staining (Figure 2E), suggesting impaired commitment of hematopoietic progenitors to the T cell lineage [33], [34]. Furthermore, thymocyte development was delayed in *Foxn1^R/R^* mice early in ontogeny (Figure 2F). Thus, rather than simply resulting from reduced numbers of functionally normal TECs, the hypoplastic *Foxn1^R/R^* phenotype was characterized by impaired TEC functionality.

**Figure 3:**
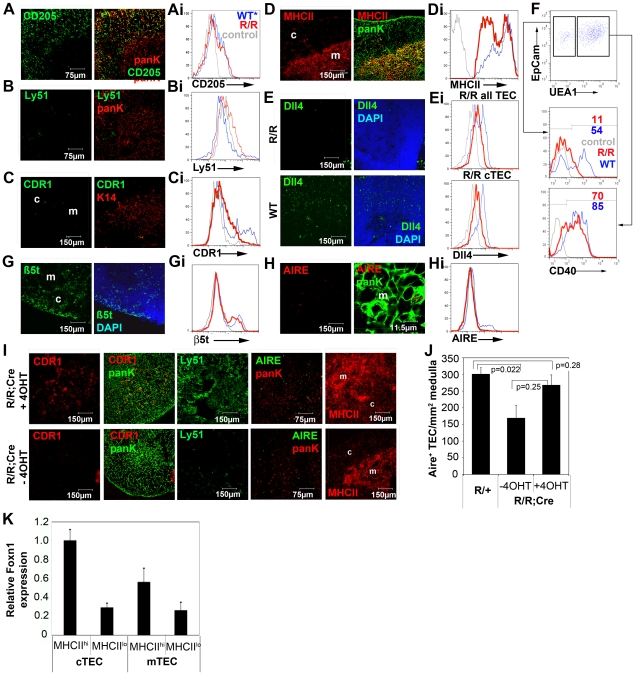
Reduced Foxn1 expression results in impaired development of both medullary and cortical TEC in postnatal *Foxn1^R/R^* thymi. (A-G, Ai-Hi) Immunohistochemical (A-E,G,H) and/or flow cytometric (Ai-Hi, F) analysis of postnatal *Foxn1^R/R^* thymi showing anti-CD205, Ly51, CDR1, anti-Aire, anti-K14, anti-MHC Class II (MHCII), anti-Dll4, anti-ß5t, anti-PanK and anti-CD40 staining. (A–E,G,H) Right panels show co-stains of left panels, right Aire panel shows detail from left panel. Scale bars 150 µm except for anti-CD205 and Ly51, which are 75 µm and Aire right panel which is 11.9 µm. (Ai-Hi, F) Plots show data after gating against CD45^+^ and Ter119^+^ and for EpCam^+^ cells. Lower panel in Ei shows data after gating on CD45-EpCam^lo^ cells (i.e. cTEC). Red lines, *Foxn1^R/R^*; blue lines, WT (*Foxn1^R/+^* rather than WT is shown for Ly51, and is indistinguishable from WT); grey lines, isotype control. All analyses are on 6 week old thymi except E and Ei which are on postnatal day 10-11 thymi. Data shown are representative of at least three independent experiments, except (F), n = 1. (I) 4 week old *Foxn1^R/R^;ROSA26^CreErt2^* mice were injected intraperitoneally with 1.5 mg 4-hydroxy tamoxifen (4OHT) and sacrificed 2 days later; bottom panels show un-injected littermate controls. Images show immunostaining with CDR1, anti-pancytokeratin, Ly51, anti-MHC Class II and anti-Aire. Data are representative of at least 3 mice for each condition. (J) 6 week old *Foxn1^R/R^;ROSA26^CreErt2^* (R/R) thymi were injected with 1.5mg 4OHT or carrier only and sacrificed 2 days later. Controls were provided by uninjected *Foxn1^R/+^;ROSA26^CreErt2^* (R/+) littermates. Frozen sections were stained with anti-Aire and anti-PanK, as in (I), and the numbers of Aire^+^ TEC per mm^2^ of medullary area were determined by counting for three medullary areas from two sections from each of three mice for each condition. Untreated *R/R* versus *R/+,* p = 0.022; 4OHT-treated *R/R* versus *R/+,* p = 0.28; 4OHT-treated *R/R* versus untreated *R/R*, p = 0.025. (K) Mature and immature postnatal cTEC (EpCAM^+^Ly51^+^MHC Class II^hi^ and EpCAM^+^Ly51^+^MHC Class II^lo^ cells respectively) and mTEC (EpCAM^+^Ly51^-^MHC Class II^hi^ and EpCAM^+^Ly51^-^MHC Class II^lo^ cells respectively) were purified from 4-8 week old WT C57BL/6 mice by flow cytometry and the relative *Foxn1* mRNA expression levels in each cell population determined by QRT-PCR. *Foxn1* is expressed at higher levels in WT cTEC than WT mTEC, and at higher levels in mature than in immature TEC in both compartments. *Foxn1* expression levels are shown relative to *alpha-tubulin* and are normalized to MHC Class II^hi^ cTEC. Data shown are the mean of two technical replicates and are representative of three independent experiments. Error bars show SD.

Interrogation of TEC subset distribution and marker expression in the *Foxn1^R/R^* thymus revealed apparently normal morphologies for medullary and cortical TEC (mTEC and cTEC respectively), and normal expression patterns for cytokeratin (K) 5, K8 and CD205 (Figure 2B, Figure 3A and 3Ai), indicating the presence of cortical and medullary compartments. Further analysis however revealed abnormal gene expression patterns in both compartments.

The cTEC marker CDR1 is acquired only postnatally in cTEC and thus represents a terminal differentiation marker for this TEC sub-lineage. Ly51 is another commonly used marker that is cTEC-specific within the thymus. Expression of both of these markers was severely down-regulated in *Foxn1^R/R^* thymi (Figure 3B-3Ci).

MHC class II, Cathepsin L and Dll4 are TEC markers with known functions in thymocyte development [11], [33]–[37]. MHC Class II staining on *Foxn1^R/R^* cTEC was dramatically reduced compared to *Foxn1^R/+^* and WT littermates (Figure 3D and 3Di), and similarly, *Cathepsin L* expression was down-regulated in *Foxn1^R/R^* cTEC (Figure S3). Dll4 expression was dramatically reduced on both fetal and postnatal *Foxn1^R/R^* TEC compared to aged-matched WT controls (Figure 3E and 3Ei, see also Figure S3). These data provide a possible explanation for the altered thymocyte subset distributions present in *Foxn1^R/R^* mice.

CD40 is expressed on TEC and thymic dendritic cells and is required for medullary development [38], [39]. Expression of CD40 was also substantially down-regulated on postnatal *Foxn1^R/R^* cTEC but was largely unaffected on mTEC (Figure 3F). Expression of the recently identified cTEC-specific thymic proteasome subunit ß5t [40] was affected only slightly if at all (Figure 3G and 3Gi; proportion of TEC that are ß5t^+^: *Foxn1^R/R^*, twenty four percent; WT, twenty four percent).

Aire is a key regulator of thymus function, and within the thymus is specifically expressed in a subset of mTEC. Significantly fewer Aire^+^ mTEC were present in *Foxn1^R/R^* compared to WT thymi (Figure 3H and 3Hi).

Collectively, these data established that differentiation of both cortical and medullary TEC was impaired in the postnatal thymus of *Foxn1^R/R^* mutants. In particular, since cTEC morphology appeared normal but several cTEC-specific markers were severely down-regulated in *Foxn1^R/R^* thymi, they strongly suggested the hypothesis that terminal differentiation was blocked in the cTEC sub-lineage.

### Reversion to wild-type Foxn1 expression levels in *Foxn1^R/R^* mice leads to rapid acquisition of terminal differentiation phenotypes in cTEC and mTEC

To test whether this hypothesis was correct, or alternatively whether the postnatal *Foxn1^R/R^* phenotype reflected the absence of a normal cTEC population and/or overgrowth of a normally minor TEC population, we bred a constitutively expressed tamoxifen inducible Cre recombinase allele, *ROSA26^CreERt2^* [41], onto the *Foxn1^R/R^* background. We then tested the outcome of reverting the *Foxn1^R^* allele via Cre-mediated excision of the LoxP-flanked cassette, such that WT levels of *Foxn1* mRNA expression were restored. Thus, adult *Foxn1^R/R^*; *ROSA26^CreERt2^* mice were injected with 4-hydroxy tamoxifen (4OHT) to induce Cre recombinase activity. Two days after 4OHT injection, *Foxn1* mRNA expression was restored to at least WT levels (Figure S3) and Ly51, CDR1 and MHC Class II staining was observed in a high proportion of cTEC (Figure 3I, see also Figure S3). *Dll4* mRNA expression was also up-regulated by two days post-injection, as was expression of both *CCL25* and *Cathepsin L* mRNA (Figure S3). Since the half-life of cTEC has been established as 10-14 days [42] these data cannot be explained by proliferation of a previously inhibited cell type followed by differentiation, but rather must reflect phenotypic conversion of existing cells. They are therefore consistent with release of a block in terminal differentiation. The proportions of Aire^+^ mTEC were also restored to WT levels in this experiment (Figure 3I, 3J).

Collectively, these data reveal two important and previously unreported roles of Foxn1. First, although the requirement for Foxn1 for postnatal TEC maintenance has recently been demonstrated [26], this is the first report of a role for Foxn1 in regulating the differentiation of postnatal TEC. Our data establish the requirement for this transcription factor for terminal differentiation of both cTEC and Aire^+^ mTEC in the postnatal thymus. Since the *Foxn1^R/R^* phenotype is most severe in the cortical compartment, they further suggest differential requirements for Foxn1 in the postnatal cortical and medullary TEC compartments. Consistent with this idea, QRT-PCR analysis indicated higher levels of *Foxn1* mRNA in cortical than medullary TEC in the normal postnatal thymus (Figure 3K). Second, they also establish that Foxn1 regulates TEC-specific expression of MHC Class II, Dll4, CD40 and Cathepsin L, which are all critically required in TEC for execution of the T cell development programme. While this regulation may be either direct or indirect, it appears to be lymphocyte-independent, as all thymocyte subsets are present in *Foxn1^R/R^* thymi.

### Generation of an allelic series expressing graded levels of Foxn1 mRNA

**Figure 4:**
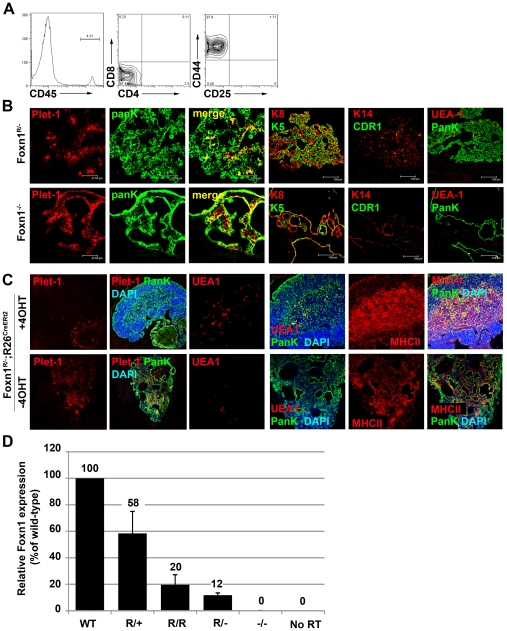
Quantitiative analysis of the *Foxn1^R^* allele. (A) Flow cytometric analysis of thymocyte development in neonatal *Foxn1^R/-^* mice. Representative plots are shown for anti-CD45, anti-CD4 versus anti-CD8 and anti-CD44 versus anti-CD25, after gating on CD45^+^ cells. *Foxn1^R/-^* thymi fail to support the maturation of immature thymocyte progenitors. Note that the tissue dissected in *Foxn1^R/-^* mice contains non-thymic tissue as well as the thymic rudiment due to the difficulty of identifying the thymic rudiment in postnatal *Foxn1^R/-^* mice. (B) Immunohistochemical analysis of postnatal *Foxn1^R/-^* and *Foxn1* ^-/-^ thymi showing Plet-1, CDR1, K14, K5, K8 and UEA-1 and PanK staining. Images shown are representative of at least three individual mice for each genotype. Scale bars 47.62 µm for Plet-1/PanK and 150 µm for K5/K8, K14/CDR1 and UEA1/PanK. (C) 2-4 month old *Foxn1^R/-^;ROSA26^CreErt2^* mice were injected intraperitoneally with 1.5 mg 4-hydroxy tamoxifen (4OHT) or carrier, and sacrificed 7 weeks later. Thymi were analyzed with the markers shown. MHCII, MHC Class II. (D) Relative *Foxn1* expression levels were determined by QRT-PCR analysis of E12.5 Plet-1^+^ TEC using the Sybr-Green method. *Foxn1* expression was normalized to the geometric mean of three housekeeping genes. Numbers show mean expression level relative to WT. (A-C) Data shown are representative of at least three independent experiments. (D) WT, *Foxn1^R/+^* and *Foxn1^R/R^*, data are representative of at least three independent experiments; *Foxn1^R/-^* and *Foxn1^-/-^*, n = 1.

We next crossed *Foxn1^R/R^* mice with mice carrying the null allele, *Foxn1^LacZ^* [13]. *Foxn1^LacZ^* phenocopies the original *nude* mutation and is referred to as *Foxn1^-^* herein. Postnatal *Foxn1^R/-^* mice had small, alymphoid thymic rudiments that were devoid of any cortical or medullary organisation, and lacked peripheral T cells (Figure 4A); no hematopoietic colonization of the *Foxn1^R/-^* thymus was seen at any stage analyzed (Figure S4). Similar to *Foxn1^nu/nu^* TEC [19] postnatal *Foxn1^R/-^* TEC were predominantly Plet-1^+^ and co-expressed Keratin 5 and Keratin 8, with occasional cells expressing the cortical and medullary TEC markers CDR1 and UEA1 respectively (Figure 4B). However, unlike the hairless *nu/nu* or *Foxn1^-/-^* mice, *Foxn1^R/-^* mice produced an overtly normal coat of hair up to six weeks of age, after which they showed sporadic hair loss. Reactivation of *Foxn1* by tamoxifen-mediated induction of Cre recombinase activity in postnatal *Foxn1^R/-^*; *ROSA26^CreERt2^* mice up to at least four months old resulted in development of an organized, functional thymus containing all major cortical and medullary TEC sub-types (Figure 4C), indicating that thymic epithelial progenitor cells can persist for prolonged periods *in vivo* in the absence of wild-type levels of Foxn1, and demonstrating that expression of SV40Tag in these cells did not affect their differentiative capacity.

The differences observed between *Foxn1^R/-^* and *Foxn1^-/-^* mice suggested that further functions of Foxn1 might be revealed by analysis of an allelic series. We therefore established a series of six strains comprising all possible combinations of *Foxn1^-^*, *Foxn1^R^* and WT alleles. Precise determination of *Foxn1* mRNA expression levels across this series relative to wild type was achieved by analysis of a defined TEC population, E12.5 Plet-1^+^ TEC (Figure 4D). In E12.5 Plet-1^+^ TEC, the *Foxn1^R^* allele expressed approximately twenty percent of WT Foxn1 mRNA levels (Figure 4D).

### Foxn1 is not required for initial divergence of the medullary TEC sub-lineage

The postnatal *Foxn1* null phenotype is characterized by the presence of linear aggregates of cells that express markers associated with the earliest thymus-restricted epithelial progenitor cells found in ontogeny [19], suggesting that, in the absence of Foxn1, TE lineage progression might be blocked at the earliest progenitor stage [19], [13]. This idea was supported by the recent demonstration of a persisting common TEPC in postnatal *Foxn1* ^-/-^ mice [8]. However, the possibility remains that more restricted precursors arise in the absence of Foxn1 but are not maintained later in ontogeny.

**Figure 5:**
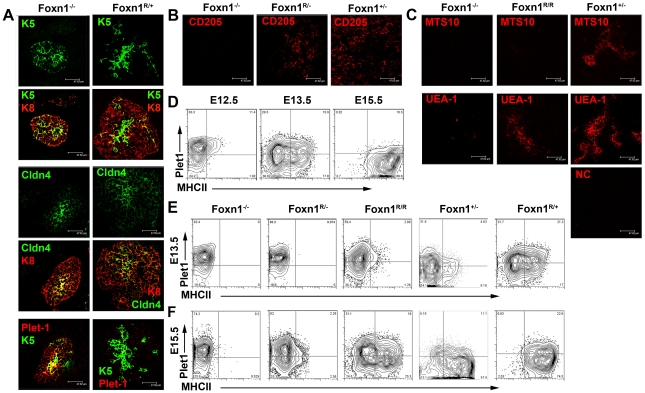
Divergence of the mTEC lineage is Foxn1-independent. Images show representative sections from (A) E13.5 thymic primordia after staining for K5 and K8, Cldn4 and Plet-1. (B,C) E15.5 thymic primordia after staining for CD205 and panK (B) or MTS10, UEA-1 and panK (C). Scale bars 47.62 µm. Genotypes of embryos are as designated. Note that the medullary lineage markers Cldn4, K5 and UEA1 are expressed in *Foxn1^-/-^* thymi. Bottom right image in (C) shows negative control (NC) for UEA1 stain (Streptavin-Alexa647). Data shown are representative of at least three independent experiments. (D-F) Plots show staining of (D) wild type thymic primordia from E12.5, E13.5 and E15.5 mice, (E) E13.5 and (F) E15.5 mice of the genotypes shown after staining with anti-Plet-1 (MTS20) and anti-MHC Class II (MHCII). All plots show data obtained after gating on EpCAM^+^ cells. The data shown for *Foxn1^-/-^*, *Foxn1^+/-^*, and E13.5 *Foxn1^R/-^* mice are representative analyses of individual thymic primordia, all other plots show analyses on pooled samples from several embryos. n≥3 for all genotypes. Relative proportions of EpCam^+^Plet-1^+^ cells in *Foxn1^-/-^* and *Foxn1^R/-^* mice: E13.5: *Foxn1^R/-^*, 70.8±16.0%; *Foxn1^-/-^*, 74.3±7.6%. E15.5: *Foxn1^R/-^*, 59±7.9%; *Foxn1^-/-^*, 73.8±13.3%; *Foxn1^R/-^* versus *Foxn1^-/-^* p = 0.74 (E13.5), p = 0.06 (E15.5).

To determine the stages in early thymus ontogeny at which *Foxn1* is required, we analyzed all strains of the allelic series (Figure 5) using marker combinations that identify early cortex (CD205 and K5^lo/-^) and early medulla (Claudin [Cldn] 4, UEA1, K5^hi^ and MTS10) [10], [11], [43], [44]. Plet-1 [5] was also included as an indicator of founder/early progenitor cell status. We focussed on the E13.5 and E15.5 time points since at these stages the TE is undergoing active patterning into cortical and medullary compartments [45]. Notably, functional analyses have shown that the Cldn4^hi^ cells present at E13.5 in the WT thymic primordium are medullary TE lineage-restricted precursors [10].

By E13.5 WT, *Foxn1^+/-^* and *Foxn1^R/+^* thymi contained central K5^hi^Cldn4^hi^ clusters surrounded by K5^lo/-^K8^+^Cldn4^lo/-^ regions (Figure 5A), as previously reported for WT thymi [3], [10], [45]; this central Cldn4^hi^ region has previously been demonstrated to correspond to prospective medulla [10]. These data thus confirmed the normal progression of TEC lineage development in *Foxn1^R/+^* mice.

K5^hi^Cldn4^hi^ and K5^-^Cldn4^lo/-^ regions were also present in the thymic primordia of *Foxn1^-/-^* mice at both E13.5 and E15.5 (Figure 5A; Figure S5). These data establish that divergence of the mTEC lineage from the common TEPC occurs in the absence of *Foxn1* and is thus *Foxn1*-independent, in contrast to the existing model of TE lineage development.

cTEC and mTEC differentiation was further probed using markers expressed at various intermediate stages of TEC development. Consistent with recent reports, neither CD205 [11] nor ß5t [40], [46] were detected in *Foxn1^-/-^* mice at any stage analyzed, confirming the dependence of the cortical sub-lineage on Foxn1 at least from the onset of expression of these markers. However, a CD205^+^ population was detectable by E15.5 in all other strains tested, including *Foxn1^R/-^* mice, demonstrating that only low levels of *Foxn1* mRNA expression are required to promote the early stages of cTEC development (Figure 5B).

By E15.5, UEA1^+^ and MTS10^+^ mTEC were present in all strains in the allelic series that express ≥50% WT levels of *Foxn1* mRNA (*Foxn1^+/-^*, *Foxn1^R/+^* and *WT* thymi). However, while strains that expressed ≤20% WT Foxn1 mRNA all contained some UEA1^+^ TEC, MTS10 expression was not detected (shown for *Foxn1^-/-^, Foxn1^R/R^* and *Foxn1^+/-^*, Figure 5C). We note that UEA1 staining was present only on rare cells in the *Foxn1^-/-^*and *Foxn1^R/-^* thymic primordium, but that this staining was consistent and above background. Since MTS10 staining was detected as normal on postnatal mTEC in *Foxn1^R/R^* mutants, but was only observed on very rare cells in postnatal *Foxn1^R/-^* and *Foxn1^-/-^* thymi, we conclude that mTEC differentiation beyond the earliest mTEC sub-lineage progenitor state is blocked in the absence, and delayed at intermediate levels, of *Foxn1* expression.

Collectively, the data described above indicate that *Foxn1* is not required for divergence of the mTEC sub-lineage from the common TEPC but is required subsequently, in a dosage dependent manner, to promote TEC differentiation. Of note is that high level Plet-1 expression is maintained in *Foxn1* ^-/-^ TECs, supporting the conclusion that these cells remain blocked in the earliest differentiation state despite having undergone lineage divergence (Figure S5).

### Low-level Foxn1 expression enables exit from the earliest sub-lineage TEPC states in the fetal thymus but is unable to sustain lineage progression

The above analysis suggested that specific intermediate states of TEC development are dependent on discrete levels of Foxn1 expression. To further test this notion, we used flow cytometry to provide quantitative evaluation of TEC differentiation status in the different allelic variants at defined time-points. For this we analyzed representative markers of the founder TE cell-state (Plet-1 [3], [5]) and functional maturation (MHC Class II [47]); TEC were positively identified using EpCam in all analyses.

In order to identify intermediate phenotypes that occur during normal TEC lineage progression we initially analyzed expression of these markers in WT mice at E12.5, E13.5 and E15.5 (Figure 5D). This demonstrated that normal TEC lineage progression is characterized by the down-regulation of Plet-1 coupled with acquisition of MHC Class II expression.

We then analyzed the allelic series. This revealed that TEC differentiation was delayed when Foxn1 expression was reduced, and that the severity of the delay was inversely proportional to *Foxn1* mRNA level. In *Foxn1^R/R^* mice the TEC subset profiles at E13.5 and E15.5 were equivalent to those of WT mice at E12.5 and E13.5 respectively (Figure 5E, 5F). Thus, normal TEC differentiation occurred in the *Foxn1^R/R^* strain, but with delayed kinetics.

The two functionally athymic strains (*Foxn1^R/-^* and *Foxn1^-/-^*) each exhibited TEC subset profiles consistent with a severe early block in TEC differentiation. Unexpectedly, the severity of this block differed between these two strains. In *Foxn1^-/-^* thymi, the ratio of Plet-1^+^ to Plet-1^lo/-^ TEC was maintained between E13.5 and E15.5 and was similar to that observed at E12.5 during normal development (determined based on comparison of the mean percentage of Plet-1^+^ and Plet-1^lo/-^ cells as shown in Figure 5E, 5F legend). However, in *Foxn1^R/-^* thymi, the proportion of Plet-1^lo/-^ TEC increased from twenty six percent at E13.5, comparable to E13.5 *Foxn1^-/-^* thymi, to forty one percent at E15.5 (see mean percentages provided in Figure 5E, 5F legend). This suggested that by E15.5, at least some *Foxn1^R/-^* TEC had been able to exit from the earliest progenitor state and enter the TEC differentiation programme, but that *Foxn1* ^-/-^ TEC could not make this transition. This conclusion was supported by analysis of the differentiation marker MHC Class II, which was expressed by a small population of Plet-1^+^ and Plet-1^lo/-^ cells in *Foxn1^R/-^* mice at E15.5, but was virtually undetectable in *Foxn1^-/-^* mice (Figure 5F). Importantly, induction of MHC class II expression occurred in the absence of colonizing haematopoietic cells (Figure S4). Thus, while TECs in the *Foxn1^-/-^* thymus remained in an undifferentiated state apparently equivalent to the earliest progenitor stages of each TEC sub-lineage, *Foxn1^R/-^* TECs could initiate the very early events associated with TEC lineage differentiation but could not progress beyond this initial differentiation step.

### Foxn1 controls allocation into or maintenance of the differentiated TEC compartment

Collectively, the above data establish that Foxn1 is required for progression through successive stages of both cTEC and mTEC differentiation. To test whether Foxn1 might also regulate TEC proliferation and/or survival, we also quantified both founder/early progenitor cells of the lineage (Plet-1^+^MHC Class II^–^ TEC) and cells that were further into the TE differentiation programme (Plet-1^-^ TEC) for each allelic variant.

**Figure 6:**
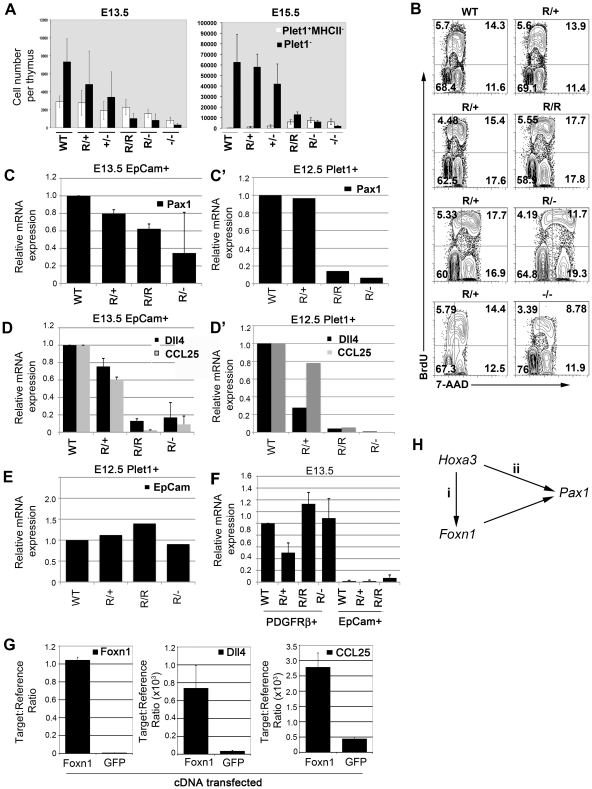
Foxn1 is required for exit from the earliest TEC progenitor states and regulates the size of the differentiated TEC compartment during ontogeny. (A) Absolute numbers of Plet-1^+^ EpCam^+^MHC Class II^-^ (MHCII)(open bars) and Plet-1^-^EpCam^+^ (black bars) cells per thymus at E13.5 and E15.5 for each genotype. Data shown are representative of at least three independent experiments. Error bars show SD. (B) Cell cycle analysis of Foxn1 allelic variants. Images show flow cytometric analysis of E15.5 WT, *Foxn1^R/+^*, *Foxn1^R/R^*, *Foxn1^R/-^* and *Foxn1^-/-^* thymi using anti-BrdU-APC and 7-AAD 1 hour after injection with BrdU. Plots are representative plots for each genotype after gating on EpCam^+^ cells; pairs of plots show data collected on the same day. (C,D,F) QRT-PCR analysis of E13.5 TEC (EpCam^+^ cells) or E13.5 thymic mesenchymal cells (PDGFRalpha^+^ cells) for the genes shown. Data are normalized to *alpha-tubulin* and shown relative to E13.5 WT EpCam^+^ cells (C,D) or E13.5 WT PDGFRalpha^+^ cells (F) and show the means from two (*Pax1*) or three (*Dll4, CCL25, Fgf2*) biological replicates for each gene. Data for all genes are representative of at least three biological replicates performed using either the Lightcycler or Fluidigm platforms. Error bars show SD. (C’,D’,E) QRT-PCR analysis of E12.5 Plet1^+^ TEC for the genes shown. Data are normalized to *EVA* [64] and shown relative to E12.5 WT Plet1^+^ cells. Data show mean of three technical replicates from one experiment on RNA from pooled thymi for each genotype. *CCL25* and *Dll4*, data are representative of two independent analyses using different analysis platforms. (G) QRT-PCR analysis of mouse embryonic stem cells after transient transfection with either *Foxn1* or *GFP*. Data show *Foxn1*, *Dll4* and *CCL25* expression levels relative to *ß actin* and show means of two (Foxn1) or three (Dll4, CCL25) biological repeats. Error bars show SD. Dll4, p = 0.009 (Foxn1 over-expression versus GFP over-expression); CCL25 p = 0.038 (Foxn1 over-expression versus GFP over-expression). (H) Cartoon shows regulatory network indicated by data shown.

At both E13.5 and E15.5, the absolute numbers of Plet-1^+^ cells correlated with Foxn1 dosage (Figure 6A), consistent with a role for Foxn1 in regulating proliferation of Plet-1^+^ TEC and thus potentially overall thymus size [48]. However, the most marked effect was on the numbers of Plet-1^-^ TEC present at different levels of Foxn1. At intermediate and low Foxn1 levels (i.e. ≤20% of WT mRNA levels; *Foxn1^R/R^*, *Foxn1^R/-^* and *Foxn1^-/-^* mice) the numbers of Plet-1^-^ TEC were decreased by four-fold compared to *Foxn1^+/-^* thymi at both E13.5 and E15.5. Notably, Foxn1 dosage was more important in determining Plet-1^+^ and Plet-1^-^ cell numbers than the presence or absence of SV40Tag (Figure 6A). These data establish that a threshold level of Foxn1 mRNA between that found in *Foxn1^R/R^* and *Foxn1^+/-^* TEC is required for generation/expansion of the Plet-1^-^ TEC population with normal kinetics, indicating a role for Foxn1 in regulating thymus size.

The above data could be consistent with roles for Foxn1 in the production, proliferation and/or maintenance of Plet-1^-^ TEC. To begin to discriminate between these possibilities we performed cell cycle analysis on TEC in all of the allelic variants at E15.5. Both *Foxn1^R/-^* and *Foxn1^-/-^* TEC exhibited slower cell cycle times than WT TEC (Figure 6B; note the proportions of cells in the G1/G0 and S/G2/M fractions). However, no differences in cell cycle were observed between E15.5 WT, *Foxn1^R/^* ^+^ and *Foxn1^R/R^* TEC (Figure 6B: note the proportions of cells in G0/G1, S and G2/M), establishing that the reduced Plet-1^-^ cell numbers in fetal *Foxn1^R/R^* thymi could not be explained by differences in proliferation rate. We therefore conclude that in the fetal thymus, *Foxn1* regulates allocation of cells into the differentiating Plet-1^-^ TEC compartment and/or the maintenance of Plet-1^-^ TEC. At present, we cannot distinguish between direct and indirect effects of Foxn1 in regulating fetal Plet-1^-^ cell numbers, as we also observed a significant delay in maturation of the thymic mesenchyme and immigration of mesenchymal cells into the thymic epithelium in *Foxn1^R/R^* mice (Figure S6), raising the possibility that the deficit in Plet-1^-^ TEC in these mutants at E15.5 might reflect impaired mesenchymal-epithelial cross talk.

### Foxn1 regulates effector genes with diverse roles in thymic epithelial cell development and function

In order to relate our findings to molecular control of TEC differentiation and function, we investigated the response of a suite of genes with diverse functions in thymus biology – i.e. that are known to regulate particular aspects of thymus development or are required in TEC to support T cell development - to changes in Foxn1 dosage. In this panel, we included genes known to be down-regulated or absent from the Foxn1 null thymic primordium or identified in our analysis as being regulated by Foxn1 (*Dll4* [34] and *CCL25* [49]), or with defined roles in TEC development (*Pax 1* [50]-[52]). We also included *Fgf2*, to date the only gene verified by chromatin immunoprecipitation in keratinocytes as a direct Foxn1 target [53]. Expression of each gene was analyzed at two time points. Initially, we analyzed expression in EpCam^+^ cells purified from the thymic primordium at E13.5. QRT-PCR analysis revealed that all of the genes tested except *Fgf2* showed changes in expression in response to Foxn1 levels (Figure 6C-6E and 6C’-6D’; see also Figure S3). In this population, *Dll4*, *CCL25* and *Pax1* were each expressed in proportion to *Foxn1* mRNA levels, though titrating to different threshold levels (Figure 6C, 6D). Our data indicate considerable heterogeneity among E13.5 TEC, raising the possibility that the observed expression patterns reflected altered TEC subset balances between the different allelic variants. Therefore, we also analyzed expression of these genes in a defined cell population, E12.5 Plet1^+^ TEC. The expression levels of all three genes were also proportional to Foxn1 expression in this population (Figure 6 C’ and D’), indicating that, in TEC, Foxn1 is genetically upstream of all three genes. In contrast, EpCam was expressed at comparable levels in the E12.5 Plet1^+^ TEC population in all of the allelic variants. Surprisingly *Fgf2* expression was detected in thymic mesenchyme but not in TEC, indicating that Foxn1 regulates different targets in TEC and keratinocytes (Figure 6F). To test whether Foxn1 regulation of *Dll4* and *CCL25* was direct or indirect, we over-expressed either the full-length *Foxn1* cDNA or *GFP* cDNA in a cell population that does not normally express *Foxn1* or either *Dll4* or *CCL25*. Both genes were significantly up-regulated in cells transfected with *Foxn1* but not in control samples (Figure 6G). These data thus strongly suggest that *Foxn1* directly regulates both *Dll4* and *CCL25*.

In conjunction with the data presented above showing Foxn1 regulates multiple intermediate TEC states, these data suggest that Foxn1 is a master regulator of the overall ‘thymus programme’ rather than regulating a particular aspect of TEC biology.

## Discussion

**Figure 7:**
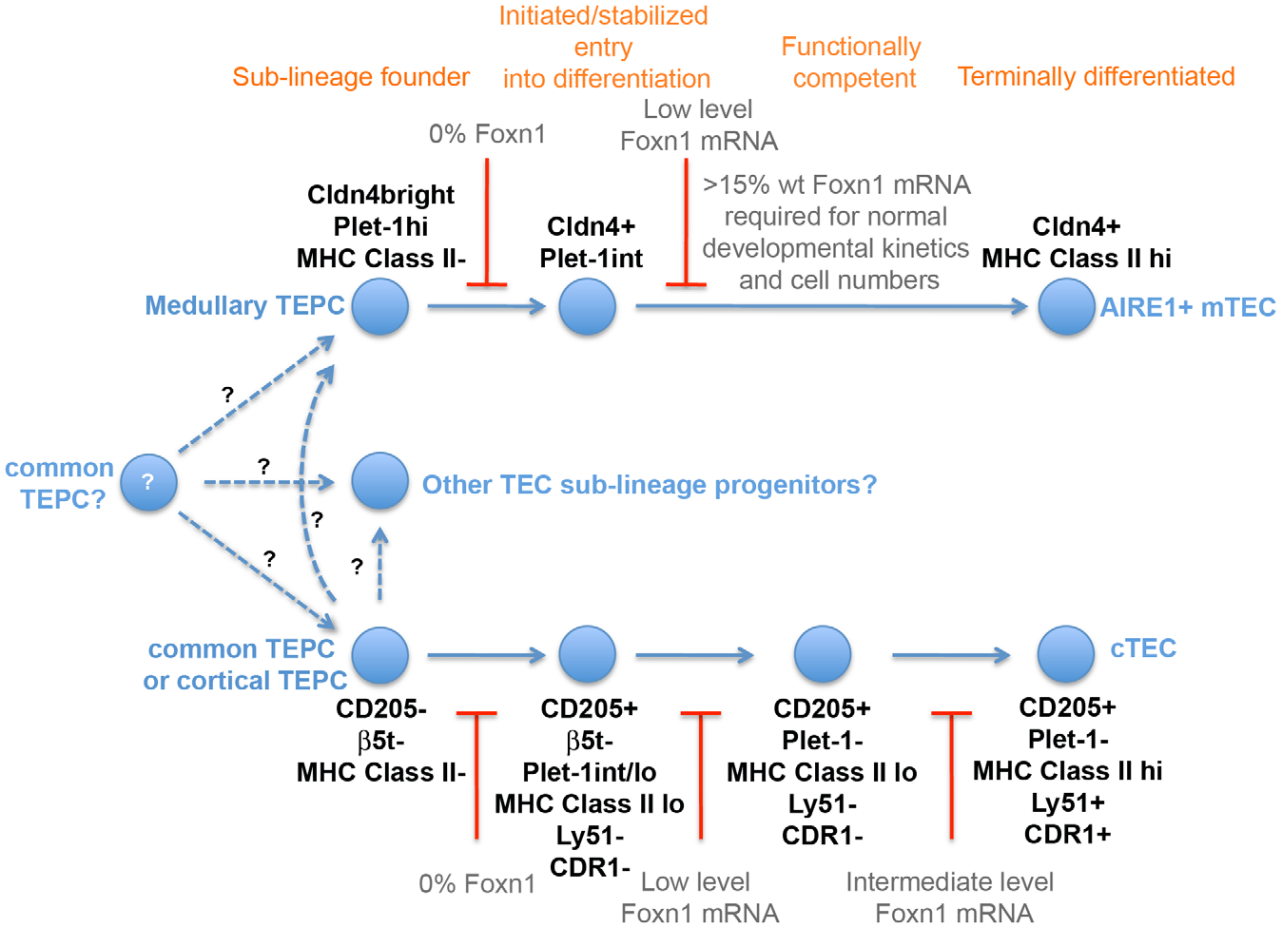
Foxn1 regulation of TE lineage development. Model of cellular hierarchies in TEC lineage progression based on the genetic data presented. The dotted lines represent lineage transitions in which the precursor-progeny relationship is currently uncertain.

We have investigated the role of Foxn1 in the TE lineage via analysis of an allelic series that expresses defined levels of *Foxn1* mRNA relative to WT. We have demonstrated that, in early ontogeny, Foxn1 is not required for divergence of the medullary TE sub-lineage from the common thymic epithelial progenitor cell; indeed we found no evidence for Foxn1 regulation of the choice between two alternative cell fates at any point in TE lineage progression. We have further demonstrated the requirement for Foxn1 for stable initiation of differentiation in the earliest TEC progenitors present in ontogeny, for subsequent progression through intermediate progenitor stages in both the cortical and medullary TEC sub-lineages, and for terminal differentiation of both cTEC and mTEC in the postnatal thymus. Taken together, these data establish that Foxn1 is required to execute the TE lineage programme at multiple stages in both the cortical and medullary TEC sub-lineages, but does not appear to regulate cell fate choice in TE lineage development. A model for Foxn1 regulation of cellular hierarchies during TE lineage development, based on the genetic analyses presented herein, is proposed in Figure 7.

In addition, we have established that Foxn1 is genetically upstream of a number of genes with known roles in TE lineage development or TEC function, specifically *Pax1*, *Dll4*, *CD40*, *Cathepsin L* and *MHC Class II*, demonstrating that *Foxn1* regulates these genes either directly or indirectly. Finally, we have confirmed and extended observations that Foxn1 is required indirectly to regulate thymic mesenchymal development [54]. These findings significantly extend understanding of regulation of thymus development and function by this key transcription factor, and pave the way for molecular dissection of these processes.

### Role of Foxn1 in TE lineage progression

With respect to TE lineage development during thymus organogenesis, our data (Figure 5) establish that Foxn1 is not required for divergence of the medullary TE sub-lineage from the common TEPC that is currently thought to exist at the basis of the TEC hierarchy [8]. This finding was unexpected, particularly given the recent demonstration of a persisting common TEPC in Foxn1 null mice [8]. Taken together with those of Bleul and colleagues, our data strongly suggest that only the common TEPC can persist in the postnatal Foxn1 null thymus and thus that Foxn1 is required directly or indirectly for TEC survival at stages subsequent to the common TEPC (including sub-lineage restricted progenitors). However, further work is required to explore this hypothesis, as an alternative possibility is that the activity of sub-lineage progenitors could not be detected in the clonal assay employed by Bleul.

Our data further establish that, subsequent to lineage divergence, Foxn1 is required in the fetal thymus for stable exit from the earliest progenitor cell state/entry into the differentiation programme leading to generation of functional TEC in both the cortical and medullary sub-lineages. Strikingly, low-level expression of Foxn1 (as found in *Foxn1^R/-^* TEC) is sufficient to initiate or stabilize entry into the TEC differentiation programme, as evidenced by acquisition of MHC Class II staining (see Figure 6), but is not sufficient to promote differentiation to a stage at which TEC in either sub-lineage are able to support T cell development. These data collectively establish that entry into the TE differentiation programme can be genetically separated from execution of the full programme (i.e. the programme leading to TEC that are functionally competent to support T cell development) based on different threshold expression levels of Foxn1. This indicates the requirement for Foxn1 for mediating both processes, and will enable dissection of the molecular role of Foxn1 at early and late differentiation stages.

We have also demonstrated, to our knowledge for the first time, that Foxn1 regulates differentiation of both cTEC and mTEC sub-lineages in the postnatal thymus. Specifically, we have shown that at intermediate levels of *Foxn1* expression (as found in *Foxn1^R/R^* TEC) differentiation of cTEC is blocked at a CD205^+^CDR1^-^ developmental stage and in addition, the number of Aire^+^ mTEC are significantly decreased. That the block in cTEC differentiation is relieved within two days of reversion to WT *Foxn1* levels provides genetic evidence for the existence of the CD205^+^CDR1^-^ intermediate stage in cortical TEC differentiation previously suggested by indirect analyses [11] and extends those findings from the fetal to the postnatal thymus. That the number of Aire^+^ mTEC is also rapidly restored to WT levels upon reversion of the *Foxn1^R^* allele suggests that differentiation of mTEC is also blocked at intermediate Foxn1 levels. From these data, we conclude that Foxn1 regulates similar mechanisms in each of the major TEC sub-lineages.

Our data further indicate MHC Class II as a direct or indirect target of Foxn1 in both fetal and postnatal TEC. Notably, TEC are unique outside the hematopoietic system in constitutively expressing MHC Class II, and the link between Foxn1 and MHC Class II expression is therefore significant.

### Foxn1 regulation of genes required for TEC development or function

Finally, we have demonstrated that Foxn1 directly or indirectly regulates a panel of genes that mediate diverse aspects of thymus development or function. These include *Pax1*, an essential mediator of TEC differentiation/survival [52]. *Pax1* is expressed in the third pharyngeal pouch from E9.5 and remains expressed throughout thymus ontogeny, being restricted to cTEC in the postnatal thymus [50]. Although regulation of this *Pax* gene is only poorly understood, from E11.0 sustained expression of *Pax1* requires *Hoxa3* [55]. Our data show, to our knowledge for the first time, that expression of *Pax1* in the thymic primordium is Foxn1-dependent. They thus place *Foxn1* and *Hoxa3* together in a network or cascade that regulates *Pax1* expression, providing the first demonstration of a genetic interaction between *Hoxa3* and *Foxn1* (Figure 6H). In this regard, it is striking that *Hoxa3^+/-^Pax1^-/-^*compound mutant mice share some phenotype characteristics with *Foxn1^R/R^* mice; both mutants have hypomorphic postnatal thymi and reduced levels of MHC class II expression on TEC [51]. As *Hoxa3* itself is expressed in TEC in all of the *Foxn1* mutants at E13.5 (data not shown), our data could support either of two alternative explanations: *Hoxa3* may regulate *Foxn1*, which in turn regulates *Pax1* expression in the thymic primordium, such that *Hoxa3* regulation of *Pax1* is *Foxn1*-dependent (Figure 6Gi), or *Hoxa3* and *Foxn1* may be required independently to maintain *Pax1* expression in the third pharyngeal pouch/early thymus primordium (Figure 6Gii). We note that since *Hoxa3* is expressed both in TEC and neural crest-derived mesenchymal cells in the thymic primordium, its regulation of *Pax1* could be either direct or indirect.

We further show that Foxn1 regulates *CCL25* and *Dll4*, which each play critical roles in thymocyte development – CCL25 regulates colonization of the fetal thymus [49] while Dll4 is the obligate Notch ligand controlling commitment of haematopoietic progenitors to the T cell lineage [34] respectively. It has previously been suggested, based on antibody staining, that although Dll4 and CCL25 are absent from the *Foxn1* null thymus [56] their expression is Foxn1-independent in TEC [57]. However, this view has been challenged in a recent report, which placed Foxn1 upstream of *dll4a* and *ccl25a* expression in *medaka* fish [58]. Our data show that in both early fetal TEC and in the postnatal thymus *Dll4* expression is proportional to *Foxn1* expression, and further demonstrate that over-expression of Foxn1 in embryonic stem cells results in expression of both *Dll4* and *CCL25*. They therefore extend the findings in *medaka* to demonstrate *Foxn1* regulation of *Dll4* and *CCL25* in the mammalian thymus, and provide strong evidence that *Foxn1* regulation of both of these target genes is direct. Reduced expression of *Dll4* in *Foxn1^R/R^* mutants also correlates with a proportional increase in intrathymic B cells, indicating that Foxn1 expression within the normal range is necessary to sustain full TEC functionality. Taken together, these data provide mechanistic insight into our findings that Foxn1 regulates multiple aspects of TEC development and function. As they demonstrate Foxn1 regulation of genes required for many different aspects of TEC biology, they suggest that this transcription factor functions a master regulator of the core TEC lineage programme. Furthermore, the finding that different Foxn1-regulated genes show distinct response patterns to changes in *Foxn1* dosage may explain why different levels of *Foxn1* are required for different functions in TEC.

Collectively, the data presented herein establish Foxn1 as powerful regulator of TEC differentiation in the fetal and adult thymus. Furthermore, they demonstrate that different threshold levels of *Foxn1* mRNA are required for different functions. Together with the recent finding that reduction of Foxn1 expression postnatally causes premature thymic involution characterized by loss of TEC [25]-[27], this suggests that Foxn1 may be required in TEC from initiation of differentiation throughout the cell's lifetime. Further work is required to determine whether it is a maintenance factor for differentiated TEC as well as having roles in proliferation and differentiation. Improved understanding of the regulation of Foxn1 in thymic development and homeostasis, and of Foxn1 regulation of its targets in different TEC sub-types, is thus likely to become a priority for strategies aimed at protecting or regenerating the thymus for therapeutic ends.

## Materials and Methods

### Ethics statement

All animal work was conducted according to UK Home Office guidelines, as established in the ANIMALS (SCIENTIFIC PROCEDURES) ACT 1986.

### Mice

*Rosa26^CreERt2/+^* [41] and *ZP3-Cre* [28] mice were maintained as homozygotes and crossed with *Foxn1^R/R^* mice as described. *Foxn1^-/-^* mice [59] were maintained as heterozygotes on a C57BL/6 background. *Foxn1^R^* mice were backcrossed onto the C57BL/6 background for at least 5 generations and subsequently maintained via intercrossing. For timed matings, noon of the day of the vaginal plug was taken as day 0.5.

### Generation of Foxn1 targeting vector

A construct containing the targeting cassette shown in Figure S1 was generated by standard molecular biology techniques and verified by sequencing. Conventional subcloning was used for the majority of cloning steps. PCR cloning using a proof-reading Taq polymerase (Roche) and TOPO-TA cloning vector (Invitrogen) was employed to insert the SV40 T antigen cassette. RecET cloning [60] was used to isolate 4 kb of the mouse Foxn1 locus from PAC367b19.

### Southern blotting

Genomic DNA was processed for Southern Blotting as described [61].

### Gene targeting and blastocyst injection

Mouse sv129/ola ES cells (line E14tg2a) were electroporated with linearized targeting vector and grown under Blasticidin selection. Three correctly targeted clones, identified by Southern analysis (Figure S1), were expanded and transfected with a Cre recombinase expression plasmid to induce deletion of the BSD cassette. Clones in which this cassette had been deleted, but which retained the rest of the targeting construct including the two remaining LoxP sites, were identified by Southern analysis, verified by sequencing and injected into C57BL/6 blastocysts to generate chimeric mice. Germ-line transmission was confirmed by genotyping for two independently targeted ES cell clones.

### Transient transfection

E14Tg2a cells were cultured in GMEM containing FCS and LIF. Transfection was carried out in 12 well plates, at a cell density of 90-95% confluence, using GeneJuice transfection reagent (Novagen). The ratio of transfection reagent to DNA was 5:1 and 1 µg of DNA was used for each well. The medium was changed 24 hrs after transfection and 1.5 µg/ml Puromycin was added to the new medium to select for transfected cells. Cells were harvested 48 hours after transfection.

### Primers

The sequences of primers used for conventional PCR and QRT-PCR are shown in Table 1 and Table 2 respectively.

**Table 1:** Primer sequences used for conventional PCR.

Gene name	Primer sequence
Foxn1 forward (Foxn1F)	5′ CATAATCCCAGCACGTGGG 3′
Foxn1 reverse (Foxn1R)	5′ GGGCGCTGAGAGAGTGAAATG 3′
Foxn1 Exon 1A forward (Ex1AF)	5′ GACGGTCGGAGCTCCTGGC 3′
Foxn1 Exon 3 reverse (Ex3R)	5′ CTCTGCTGGGAAGCTAGGC 3′
SV40TAg forward (TF)	5′ TGCCTAAAACACTGCAGGCC 3′
SV40Tag reverse (TR)	5′ TGAAATTTGTGATGCTATTGC 3′
ß actin forward	5′ GGCCCAGAGCAAGAGAGGTATCC 3′
ß actin reverse	5′ ACGCACGATTTCCCTCTCAGC 3′

**Table 2:** Primer sequences used for QRT-PCR.

Gene name	Primer sequence
alpha-tubulin forward	5′ CGGACCACTTCAAGGACTAAA 3′
alpha-tubulin reverse	5′ ATTGCCGATCTGGACACC 3′
Foxn1 forward	5′ TGACGGAGCACTTCCCTTAC 3′
Foxn1 reverse	5′ GACAGGTTATGGCGAACAGAA 3′
Dll4 forward	5′ AGGTGCCACTTCGGTTACAC 3′
Dll4 reverse	5′ GGGAGAGCAAATGGCTGATA 3′
CCL25 forward	5′ GAGTGCCACCCTAGGTCATC 3′
CCL25 reverse	5′ CCAGCTGGTGCTTACTCTGA 3′
Pax1 forward	5′ CTCCGCACATTCAGTCAGC 3′
Pax1 reverse	5′ TCTTCCATCTTGGGGGAGTA 3′
Fgf2 forward	5′ CGGCTCTACTGCAAGAACG 3′
Fgf2 reverse	5′ TGCTTGGAGTTGTAGTTTGACG 3′
Cathepsin L forward	5′ CAAATAAGAATAAATATTGGCTTGTCA 3′
Cathepsin L reverse	5′ TGTAGCCTTCCATACCCCATT 3′
EpCam forward	5′ AGAATACTGTCATTTGCTCCAAACT 3′
EpCam reverse	5′ GTTCTGGATCGCCCCTTC 3′
EVA forward	5′ TGTGCTTCCACTTCTCCTGA 3′
EVA reverse	5′ TCCACAGCTTCTGTAGGACAAA 3′

### Genotyping

Foxn1F and Foxn1R were used to detect the wild-type Foxn1 allele. TF and TR were used to detect the *SV40 T antigen* cDNA.

### Antibodies

MTS20 (IgM) and MTS24 (IgG2a), rat mAbs that recognise Plet-1 [5], and MTS10, a rat mAb that recognises medullary TEC, were kind gifts from R.L. Boyd; anti-EpCAM (G8.8, rat IgG2a, DSHB); anti-Cytokeratin 8 (Troma 1, rat IgG2a, DSHB); anti-Cytokeratin 14 (LL002, mouse IgG3 was a kind gift from E.B. Lane); anti-Cytokeratin 5 (AF138, rabbit IgG, Covance); anti-Claudin 4 (rabbit IgG was a kind gift from S. Tsukita); anti-Dll4 was a kind gift from F. Radtke; anti-CD3-FITC (145-2C11, hamster IgG1); anti-CD4-FITC or PE (H129.19, rat IgG2a); anti-CD8-FITC (53-6.7, rat IgG2a); anti-CD11b-FITC (M1/70, rat IgG2b); anti-CD11c-FITC (HL3, hamster IgG1); anti-α-CD19-FITC (1D3, rat IgG2a); anti-CD25-PE (3C7, rat IgG2b); anti-CD44-APC (1M7, rat IgG2b); anti-Ly76-FITC (Ter119, rat IgG2b); anti-CD31-FITC or purified (390, rat IgG2a); anti-CD45-APC (30-F11, rat IgG2b); anti-PDGFRalpha (APA-5, rat IgG2a); anti-SV40 T antigen (PAb100, mouse IgG1)(all Pharmingen); ERTR7 (rat IgG2a was a kind gift from W. van Ewijk); anti-Cytokeratin (rabbit IgG polyclonal, DAKO); CDR1 (Rat IgG2a, was a kind gift from B Kyewski); anti-Foxn1 C-terminal (IMG-3744, polyclonal, Imgenex); Ly51 (Clone 6C3, Rat IgG2a, Biolegend); anti-ß5t (Rabbit polyclonal, purified, IgG, MBL International); biotinlyated UEA-1 (Vector Laboratories). For detection of unconjugated primaries the following secondary antibodies were used; goat anti-rabbit IgG-alexa488; goat anti-rat IgG-alexa647; goat anti-rat IgG-alexa488; goat anti-mouse IgG-alexa647; donkey anti-rat IgG-alexa488; Streptavidin-alexa647 (all Molecular Probes); mouse anti-rat IgM-PE (G53-238, Pharmingen).

### Flow cytometry

#### Analysis

Mouse fetal thymi were processed for flow cytometric analysis as described [3]. Adult TEC were enzymatically digested to single cell suspensions as described for fetal thymi, and processed for flow cytometric analysis without further enrichment. Adult thymocytes were isolated by mechanical disruption of 5–6 week old thymi and stained with the appropriate antibodies. All staining was for 20 minutes on ice in PBS/5%FCS/5U/ml DNAseI. Data were acquired using a FACS Cailibur (BD San Jose, Ca) or Cyan (Beckman Coulter, Miami, Fl) cytometers and analyzed using Flowjo version 6.4.6 (Tree Star, Inc) software.

#### Sorting

For isolation of fetal TEC, MTS20^+^CD45^-^Ter119^-^CD31^-^PDGFRalpha^-^ and MTS20^-^CD45^-^Ter119^-^CD31^-^PDGFRalpha^-^ populations were purified; anti-EpCam was also used to positively identify TEC in some sorts (as indicated). For isolation of adult TEC, TEC were enriched as described [62] and EpCAM^+^Ly51^+^MHC Class II^hi^, EpCAM^+^Ly51^+^MHC Class II^lo^, EpCAM^+^Ly51^-^MHC Class II^hi^, and EpCAM^+^Ly51^-^MHC Class II^lo^ populations purified after gating against CD45^-^Ter119^-^ cells. Cell sorting was performed using MoFlo (Dakocytomation) and FACSAria (BD) cell sorters, to a purity of >95% for each sample. For all samples, compensations were determined using antibody capture beads (made in house by S. Monard) and Fluorescence Minus One (FMO) staining was used to determine positivity for each antibody. 7AAD was used to identify dead cells in all samples.

### Immunohistochemistry

Whole E13.5 – E15.5 embryos or adult thymi were processed for immunohistochemistry as described [2]. Isotype controls (not shown) were included in all experiments. Staining was analyzed using a Leica AOBS confocal microscope (Leica Microsystems GmbH). The images presented are either single optical sections or projected focus stacks of serial optical sections. The number of Aire^+^ TEC per mm medullary area was established by analyzing three medullary areas on each of two non-sequential sections from each of three individual mice (i.e. the number of Aire^+^ cells was counted for six different medullary areas for each of three individual mice per condition). The size of each medullary area was calculated using Adobe Photoshop and the number of Aire^+^ cells per square mm was then calculated. Statistical analysis was on the average number of Aire^+^ cells per condition.

### Tamoxifen injection

Mice were treated with a single intraperitoneal injection of 1.5 mg 4-hydroxy tamoxifen (4OHT) prepared in ethanol and diluted appropriately in Cremophor (Sigma)/PBS.

### RNA isolation

RNA was prepared using Tri-reagent and RNAeasy (both Qiagen) according to manufacturer's instructions. All samples were DNase treated.

### QRT-PCR

cDNA was prepared using the Superscript II first strand synthesis kit (Invitrogen) with Oligo-dT primers, according to manufacturer's instructions. For data shown in Figure 4, the IQ SYBR Green Supermix (Bio-Rad) was used for quantification and the relative expression level of the target genes was normalized to the geometric mean of three control genes (Hprt, Ywhaz, Hmbs). For data shown in Figure 3, Figure 6C, 6D, 6F and 6G, Figure S2 and Figure S3, relative expression levels were determined using the Roche Universal Probe Library (Foxn1, Probe 68; SV40Tag, Probe 32; alpha-tubulin, probe 58; Dll4, probe 106; CCL25, probe 9; Pax1, probe 105; Fgf2, probe 4; EpCam, probe 52; EVA, probe 100) with the Roche Lightcycler 480. Relative expression levels are shown after normalization to *alpha-tubulin* expression using Roche LC480 Relative Quantification software. Technical duplicates or triplicates were run for all samples and no RT and no template controls were included in all experiments. For data shown in Figure 6C’, 6D’ and 6E, relative expression levels were determined using the Roche Universal Probe Library (as above) using microfluidic QRT-PCR (Fluidigm). Pre-amplification was carried out using target specific primer pairs according to the manufacturer's protocol. Samples were loaded onto a BioMark 48.48 Dynamic Array (Fluidigm) and thermal cycling was performed using a BioMark instrument (Fluidigm) according to the manufacturer's protocol. Data analysis was carried out using BioMark Real-Time PCR Analysis Software v2.0 (Fluidigm) and the ΔCt method [63]. Expression levels are shown relative to WT after normalization to Epithelial V-like Antigen (EVA)[64].

### Cell cycle analysis

Pregnant female mice were injected with 1mg BrdU at E15.5 and embryos were collected 1 hour after injection. Thymi were immediately microdissected, dissociated and stained with G8.8 (Pharmingen), anti-CD45-PE, anti-BrdU-APC and 7-AAD using the Pharmingen APC BrdU Flow Kit according to manufacturer's instructions.

### Western blotting

Nuclear protein fractions were prepared using the Active Motif Nuclear Extract Kit according to manufacturer's instructions, separated by electrophoresis on a SDS-PAGE gel (Novex, Invitrogen), and processed for Western Blotting as described [5].

### Statistical analysis

Statistical analysis was performed using the one-way ANOVA test (two tailed), as appropriate for normally distributed data (normal distribution was tested using Chi^2^ goodness of fit). The alpha level is taken as 0.05. Errors shown are standard deviations throughout. Sample sizes of at least n = 3 were used for statistical analyses.

## Supporting Information

Figure S1 Generation of *Foxn1^R^* mice, related to Figure 1. (A) A LoxP flanked cassette containing the following elements: the 5′ engrailed 2 splice acceptor site (SA), the SV40TAg coding region, and internal ribosome entry site coupled to an enhanced green fluorescent protein/neomycin resistance fusion protein (IRES-EGFPneo), the CMAZ transcriptional pause and a LoxP flanked Blasticidin-S deaminase (BSD) resistance gene was inserted into intron 1b of the *Foxn1* locus of mouse ES cells by conventional gene targeting methods. E, exon. (B) Blasticidin resistant colonies were isolated and screened by Southern blotting of Nde1 digested genomic DNA using 5′ and 3′ flanking probes as indicated. Correctly targeted clones yielded 7.5kb and 5.3kb bands corresponding to the wild-type and targeted alleles respectively when screened with the 5′ probe, and 7.5kb and 9.3kb bands corresponding to the wild-type and targeted alleles respectively when screened with the 3′ probe. (C, D) To remove the BSD selectable marker targeted ES cell clones were electroporated with a Cre expression vector to allow the transient expression of Cre recombinase and thus excision of the floxed BSD cassette. Following transfection, cells were plated at clonal density, expanded and transferred to master and duplicate plates. Blasticidin sensitive colonies were screened by Southern blotting of Nde1 digested genomic DNA using the 3′ flanking probe to identify clones in which only the BSD cassette had been removed. Clones in which this event had occurred yielded an 8.3kb band in addition to the 7.5kb band corresponding to the wild-type allele. Three independent clones were identified and subsequently injected into C57BL/6 blastocysts to generate chimeras. Of four high contribution chimeras tested two produced agouti coloured offspring, indicating that the ES cells had contributed to the germline. This was confirmed by genotyping. The transgenic line, designated *Foxn1* ^R^, was back-crossed onto the C57BL/6 background and subsequently maintained as homozygotes. Genotypes are indicated above each image. Foxn1^R/R^ thymi exhibit a relative decrease in Foxn1 protein levels compared to wild-type and Foxn1^R/+^ thymi. wt, wild-type; R/+, *Foxn1* ^R/+^; R/R, *Foxn1* ^R/R^.
(TIF)

Figure S2 SV40Tag is expressed at low levels from the Foxn1 locus in *Foxn1^R/R^* TEC *in vivo*. (A) Image shows staining for SV40Tag (Tag) and pancytokeratin (PanK) in the MC2x3a (MC2x3) cell line, which was established from E12.5 TEC from *Foxn1^R/R^* mice. The line was passaged for several months before analysis. (B) Image shows staining for SV40Tag, pancytokeratin and DAPI on postnatal thymus sections from *Foxn1^R/R^* mice. No SV40Tag staining was ever detected on thymus sections, despite analyzing mice of different ages and using different staining protocols. (C) QRT-PCR analysis of cells from the MC2x3a cell line, and purified EpCam^+^ TEC from 6 week old *Foxn1^R/R^* and WT mice. Data shown are relative to *alpha-tubulin*. TEC were isolated as described in Materials and Methods.
(TIF)

Figure S3 Relative mRNA expression in postnatal WT and *Foxn1^R/R^* UEA1^-^EpCam^+^ TEC and in *Foxn1^R/R^;R26^CreERt2^* UEA1^-^EpCam^+^ TEC 2 days after 4OHT injection. Thymi from three 4-week old mice from each genotype (WT, *Foxn1^R/R^* and 4OHT-treated *Foxn1^R/R^;R26^CreERt2^*) were dissected and processed for flow cytometric cell sorting after enrichment for CD45^-^ cells by AutoMACS, as described in Materials and Methods. Since *Foxn1^R/R^* cTEC express low levels or no Ly51 and CDR1, in order to enrich for cTEC, we purified EpCam^+^UEA1^-^ cells. CD205 was not used since, in our hands, CD205 does not stain all Ly51^+^ cTEC. The purified EpCam^+^UEA1^-^ cells for each genotype were pooled and processed for QRT-PCR analysis of *Foxn1*, *Dll4*, *CCL25* and *Cathepsin L* as described in Materials and Methods. Data shown are relative to *alpha-tubulin* and are normalized to WT (n = 1).
(TIF)

Figure S4 Lack of colonisation of the fetal *Foxn1^R/-^* thymic primordium by hematopoietic cells. Images show immunohistochemical analysis of E13.5 thymi showing anti-CD45 (red) and anti-PanK (green) for each genotype as indicated. CD45^+^ cells can be seen within the thymic epithelium in *Foxn1^R/R^* mice but are reduced in number compared to *Foxn1^R/+^* controls. CD45^+^ cells in *Foxn1^R/-^* mice remain localized to peri-thymic areas and fail to migrate into the thymic epithelium. Scale bars 47.62 µm. Representative of at least three separate analyses.
(TIF)

Figure S5 Effect of *Foxn1* dosage on TEC development during ontogeny. Images show representative sections from E13.5 or E15.5 thymic primordia after staining for (A) K5 and K8, and (B) Plet-1 (MTS24) and panK. Scale bars 47.62 µm. Genotypes are as indicated. Data shown are representative of at least three independent experiments.
(TIF)

Figure S6 Regulation of thymic mesenchymal development by Foxn1. A) Images show transverse sections of E15.5 thymi stained with α-PDGFRalpha, ERTR7 and PanK as indicated. Genotypes are as shown. Immigration of ERT7^+^ mesenchyme into the thymus primordium is impaired in *Foxn1^R/R^*, *Foxn1^R/-^* and *Foxn1^-/-^* mice. Scale bars, 47.62 µm. (B) Flow cytometric analysis of whole E15.5 thymi. Plots show staining with α-PDGFRalpha and anti-PDGFRß after gating on EpCam^-^CD45^-^ cells. PDGFRalpha^+^ cells represent an increased proportion of total thymic mesenchyme in the *Foxn1* hypomorphic mutants compared to *Foxn1^R/+^* controls and the hypomorphic mutants also express higher levels of PDGFRalpha. Data shown are representative of at least three independent experiments.
(TIF)
