# Supplementary figures and images for: Foxn1 Regulates Lineage Progression in Cortical and Medullary Thymic Epithelial Cells But Is Dispensable for Medullary Sublineage Divergence
Source: PLoS Genet. 2011 Nov 3;7(11):e1002348. doi: 10.1371/journal.pgen.1002348 (PMC3207875; doi:10.1371/journal.pgen.1002348)

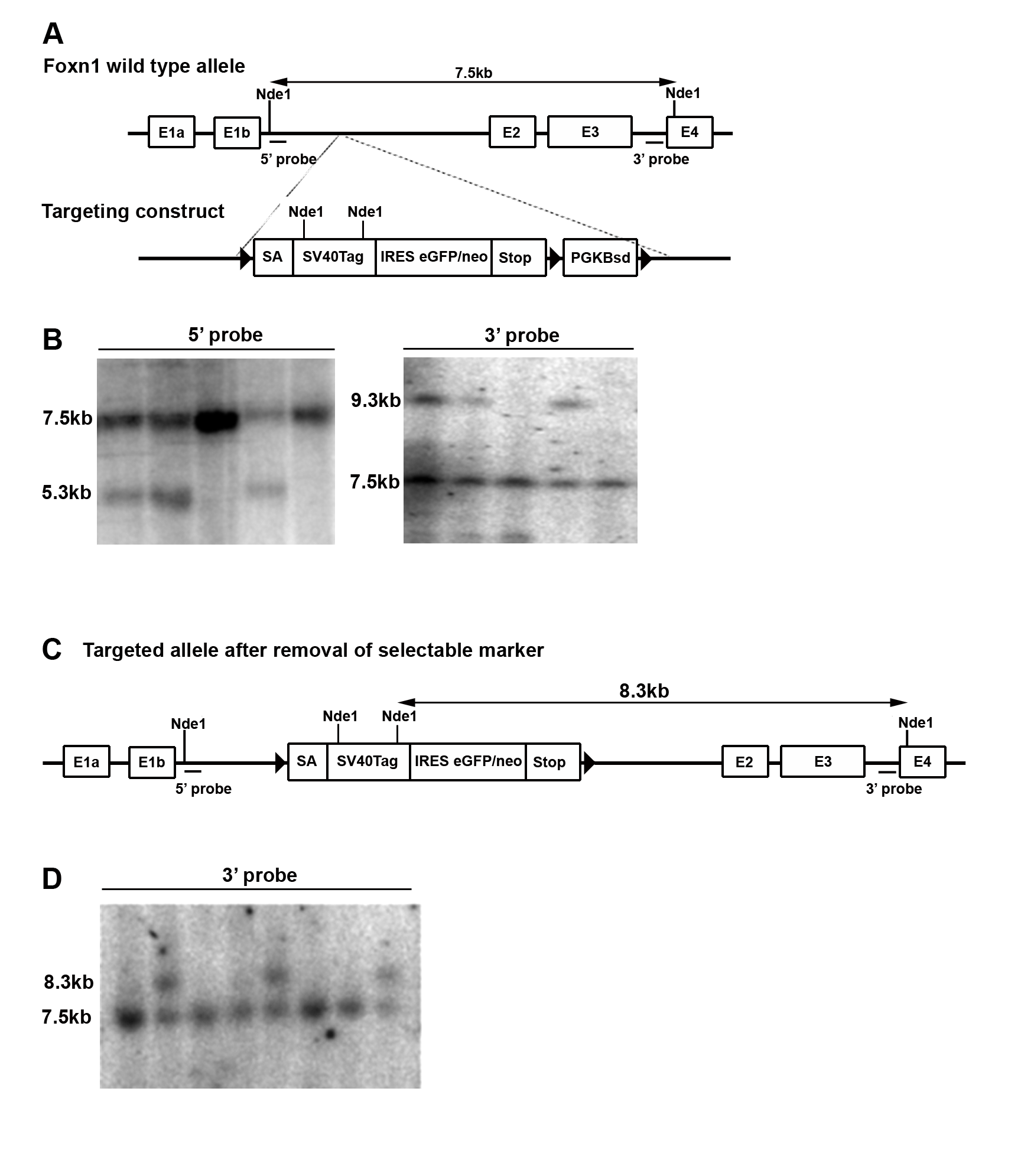

Supplement: Figure S1 — Generation of Foxn1R mice, related to Figure 1. (A) A LoxP flanked cassette containing the following elements: the 5′ engrailed 2 splice acceptor site (SA), the SV40TAg coding region, and internal ribosome entry site coupled to an enhanced green fluorescent protein/neomycin resistance fusion protein (IRES-EGFPneo), the CMAZ transcriptional pause and a LoxP flanked Blasticidin-S deaminase (BSD) resistance gene was inserted into intron 1b of the Foxn1 locus of mouse ES cells by conventional gene targeting methods. E, exon. (B) Blasticidin resistant colonies were isolated and screened by Southern blotting of Nde1 digested genomic DNA using 5′ and 3′ flanking probes as indicated. Correctly targeted clones yielded 7.5kb and 5.3kb bands corresponding to the wild-type and targeted alleles respectively when screened with the 5′ probe, and 7.5kb and 9.3kb bands corresponding to the wild-type and targeted alleles respectively when screened with the 3′ probe. (C, D) To remove the BSD selectable marker targeted ES cell clones were electroporated with a Cre expression vector to allow the transient expression of Cre recombinase and thus excision of the floxed BSD cassette. Following transfection, cells were plated at clonal density, expanded and transferred to master and duplicate plates. Blasticidin sensitive colonies were screened by Southern blotting of Nde1 digested genomic DNA using the 3′ flanking probe to identify clones in which only the BSD cassette had been removed. Clones in which this event had occurred yielded an 8.3kb band in addition to the 7.5kb band corresponding to the wild-type allele. Three independent clones were identified and subsequently injected into C57BL/6 blastocysts to generate chimeras. Of four high contribution chimeras tested two produced agouti coloured offspring, indicating that the ES cells had contributed to the germline. This was confirmed by genotyping. The transgenic line, designated Foxn1 R, was back-crossed onto the C57BL/6 background and [file pgen.1002348.s001.tif]

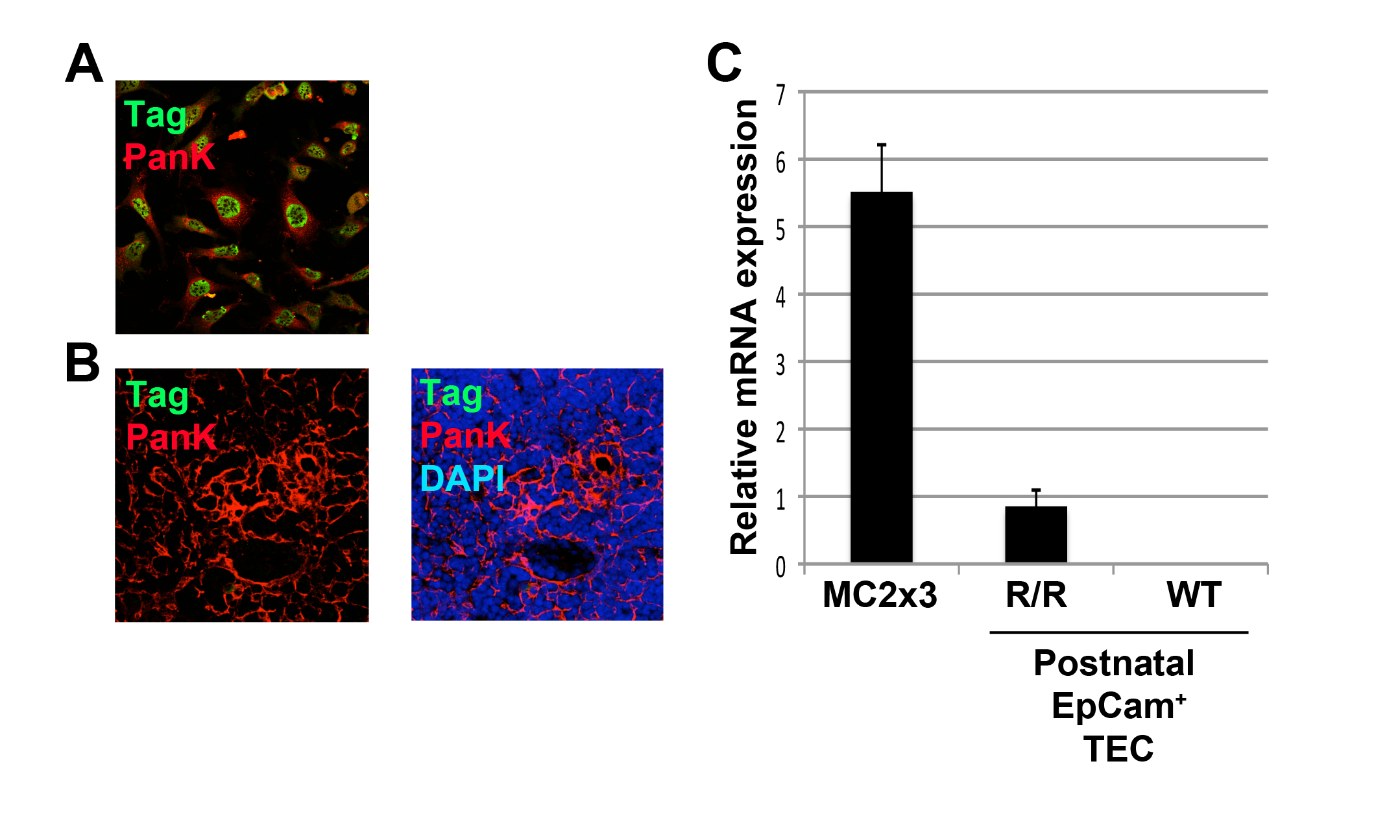

Supplement: Figure S2 — SV40Tag is expressed at low levels from the Foxn1 locus in Foxn1R/R TEC in vivo. (A) Image shows staining for SV40Tag (Tag) and pancytokeratin (PanK) in the MC2x3a (MC2x3) cell line, which was established from E12.5 TEC from Foxn1R/R mice. The line was passaged for several months before analysis. (B) Image shows staining for SV40Tag, pancytokeratin and DAPI on postnatal thymus sections from Foxn1R/R mice. No SV40Tag staining was ever detected on thymus sections, despite analyzing mice of different ages and using different staining protocols. (C) QRT-PCR analysis of cells from the MC2x3a cell line, and purified EpCam+ TEC from 6 week old Foxn1R/R and WT mice. Data shown are relative to alpha-tubulin. TEC were isolated as described in Materials and Methods. (TIF) [file pgen.1002348.s002.tif]

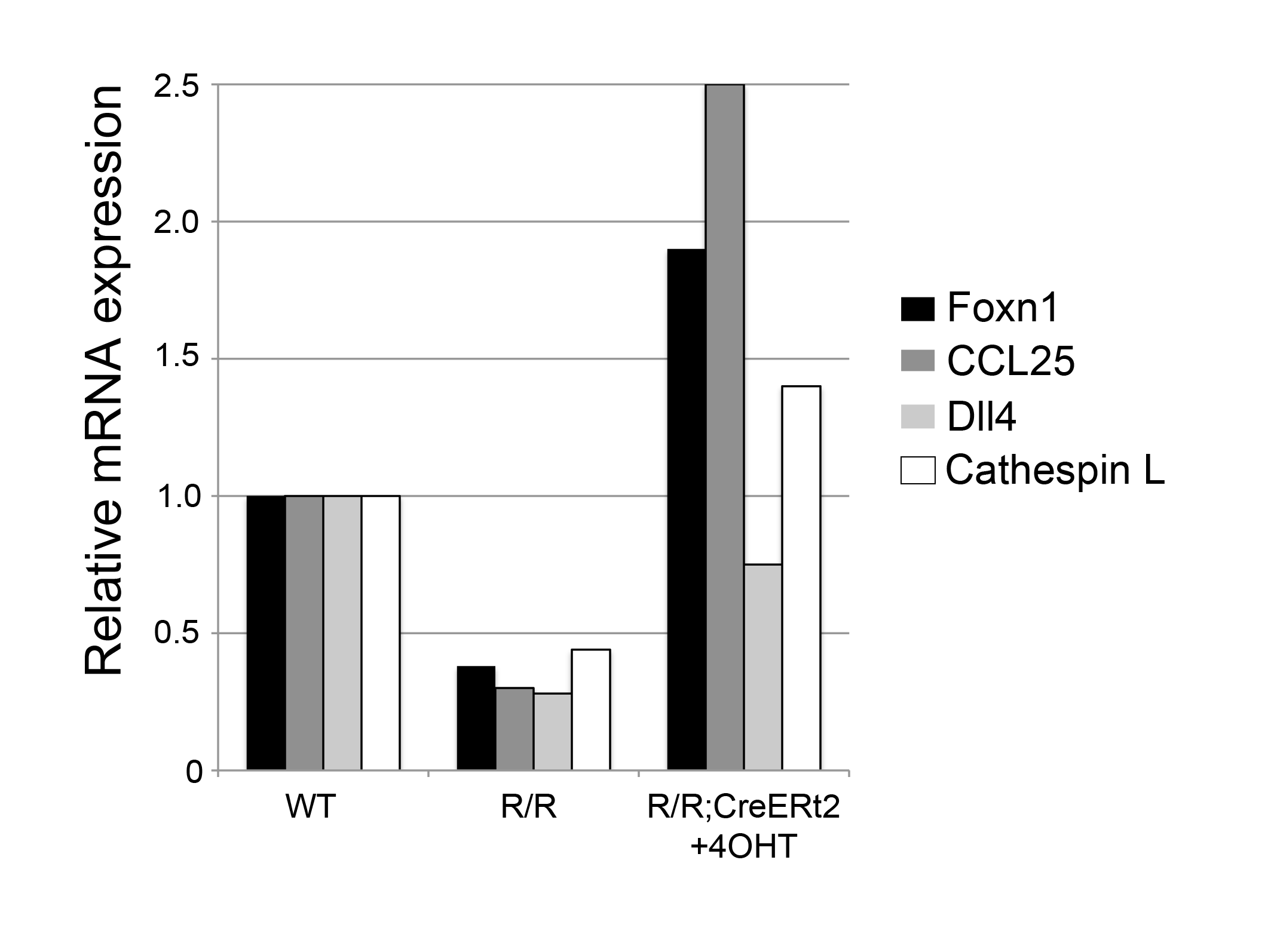

Supplement: Figure S3 — Relative mRNA expression in postnatal WT and Foxn1R/R UEA1-EpCam+ TEC and in Foxn1R/R;R26CreERt2 UEA1-EpCam+ TEC 2 days after 4OHT injection. Thymi from three 4-week old mice from each genotype (WT, Foxn1R/R and 4OHT-treated Foxn1R/R;R26CreERt2) were dissected and processed for flow cytometric cell sorting after enrichment for CD45- cells by AutoMACS, as described in Materials and Methods. Since Foxn1R/R cTEC express low levels or no Ly51 and CDR1, in order to enrich for cTEC, we purified EpCam+UEA1- cells. CD205 was not used since, in our hands, CD205 does not stain all Ly51+ cTEC. The purified EpCam+UEA1- cells for each genotype were pooled and processed for QRT-PCR analysis of Foxn1, Dll4, CCL25 and Cathepsin L as described in Materials and Methods. Data shown are relative to alpha-tubulin and are normalized to WT (n = 1). (TIF) [file pgen.1002348.s003.tif]

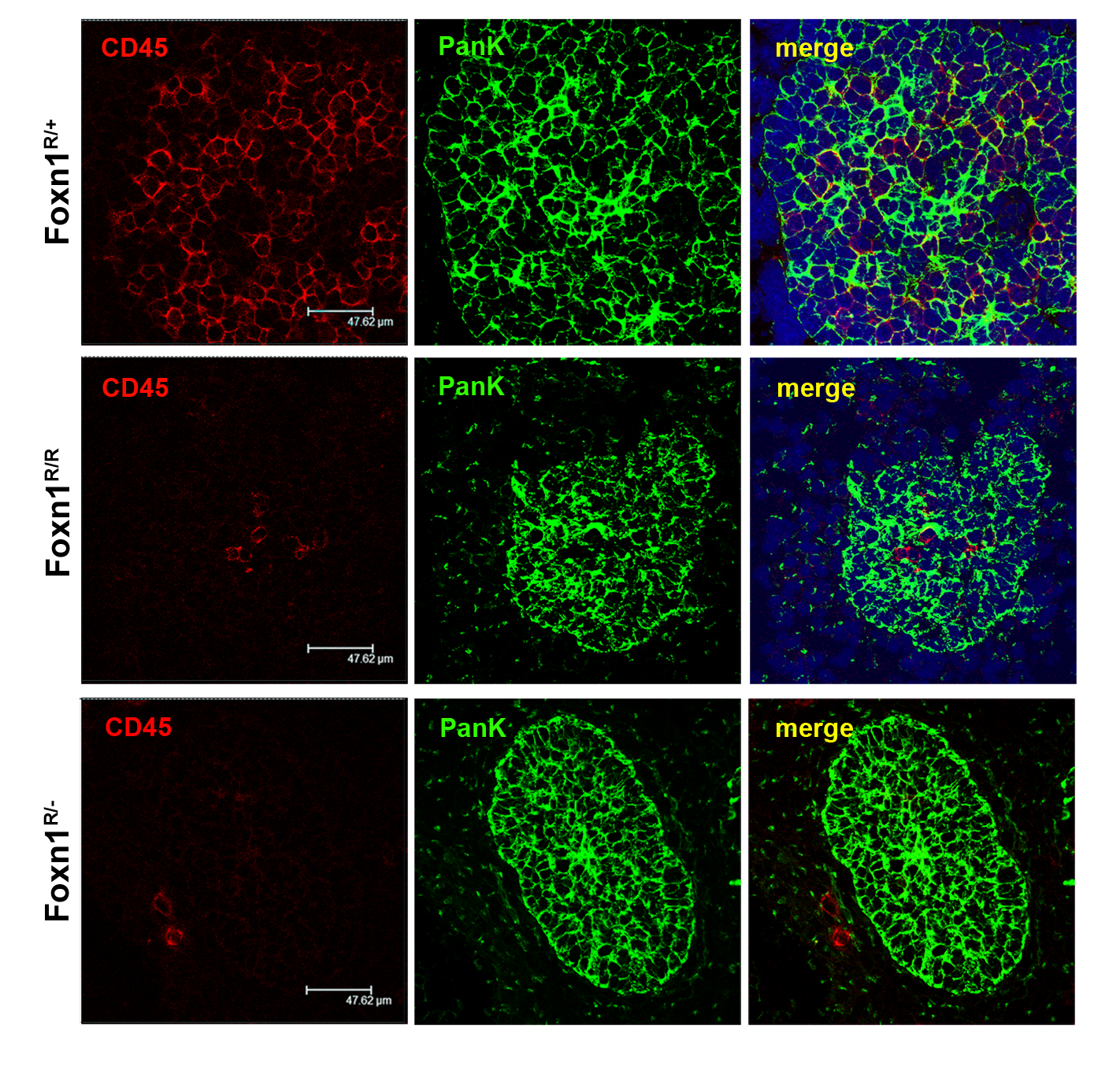

Supplement: Figure S4 — Lack of colonisation of the fetal Foxn1R/- thymic primordium by hematopoietic cells. Images show immunohistochemical analysis of E13.5 thymi showing anti-CD45 (red) and anti-PanK (green) for each genotype as indicated. CD45+ cells can be seen within the thymic epithelium in Foxn1R/R mice but are reduced in number compared to Foxn1R/+ controls. CD45+ cells in Foxn1R/- mice remain localized to peri-thymic areas and fail to migrate into the thymic epithelium. Scale bars 47.62 µm. Representative of at least three separate analyses. (TIF) [file pgen.1002348.s004.tif]

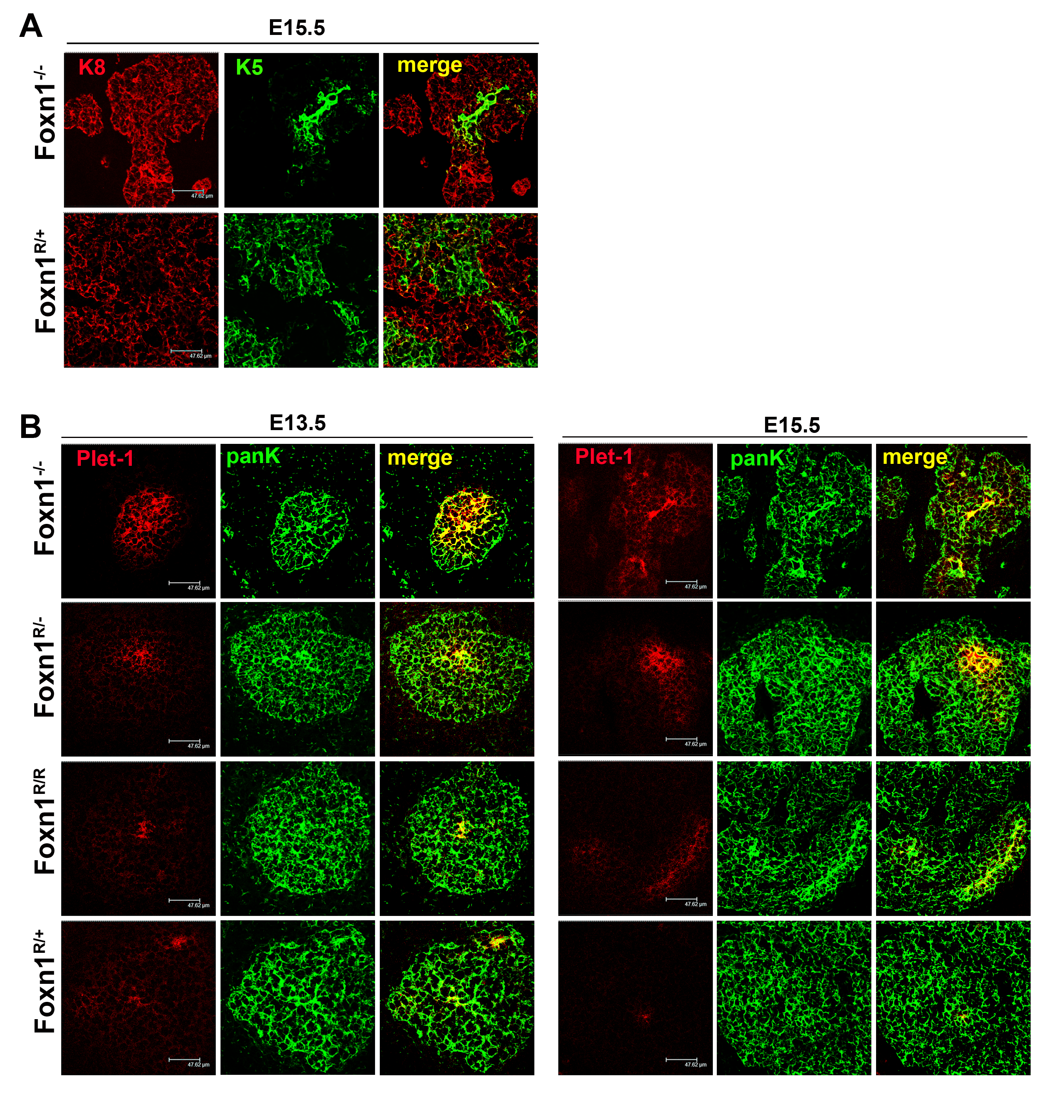

Supplement: Figure S5 — Effect of Foxn1 dosage on TEC development during ontogeny. Images show representative sections from E13.5 or E15.5 thymic primordia after staining for (A) K5 and K8, and (B) Plet-1 (MTS24) and panK. Scale bars 47.62 µm. Genotypes are as indicated. Data shown are representative of at least three independent experiments. (TIF) [file pgen.1002348.s005.tif]

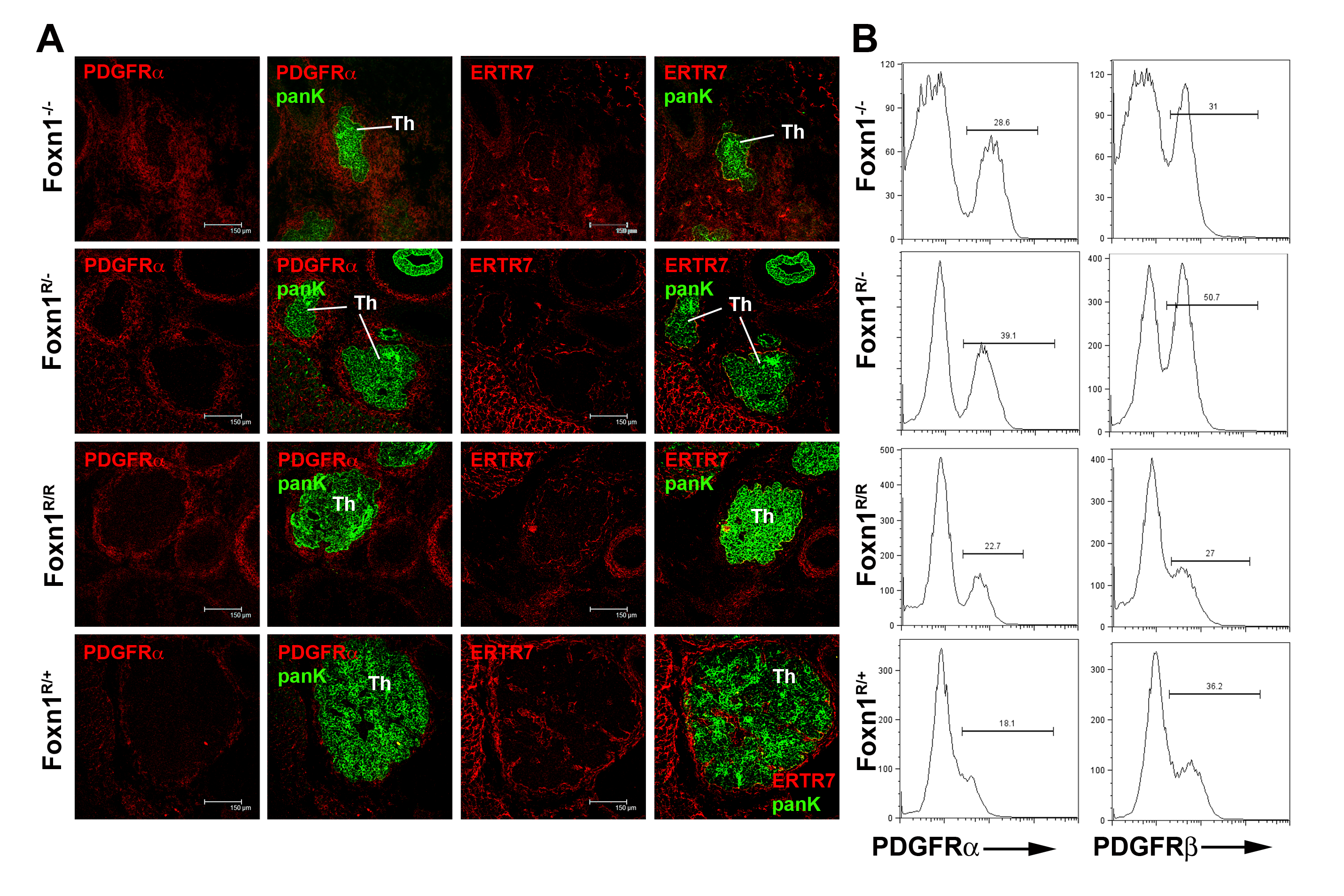

Supplement: Figure S6 — Regulation of thymic mesenchymal development by Foxn1. A) Images show transverse sections of E15.5 thymi stained with α-PDGFRalpha, ERTR7 and PanK as indicated. Genotypes are as shown. Immigration of ERT7+ mesenchyme into the thymus primordium is impaired in Foxn1R/R, Foxn1R/- and Foxn1-/- mice. Scale bars, 47.62 µm. (B) Flow cytometric analysis of whole E15.5 thymi. Plots show staining with α-PDGFRalpha and anti-PDGFRß after gating on EpCam-CD45- cells. PDGFRalpha+ cells represent an increased proportion of total thymic mesenchyme in the Foxn1 hypomorphic mutants compared to Foxn1R/+ controls and the hypomorphic mutants also express higher levels of PDGFRalpha. Data shown are representative of at least three independent experiments. (TIF) [file pgen.1002348.s006.tif]
